# Silicon Carbide Ceramics for Armor Applications: A Review of Sintering Methods and Additive Systems

**DOI:** 10.3390/molecules31071185

**Published:** 2026-04-02

**Authors:** Dauren Zhambakin, Madi Abilev, Almira Zhilkashinova, Aigerim Ichshanova, Leszek Łatka

**Affiliations:** 1 Department of Analytical, Colloid Chemistry and Technology of Rare Elements, Al-Farabi Kazakh National University, 71 Al-Farabi Avenue, Almaty 050040, Kazakhstan; zhambakin@mail.ru; 2 National Research Laboratory for Collective Use, Sarsen Amanzholov East Kazakhstan University, 34 Tridtsatoy Gvardeiskoy Divizii Street, Ust-Kamenogorsk 070002, Kazakhstan; amira_q@mail.ru; 3 Faculty of Mechanical Engineering, Wroclaw University of Science and Technology, 5 Lukasiewicza Street, 50-371 Wroclaw, Poland; leszek.latka@pwr.edu.pl

**Keywords:** armor ceramics, fracture toughness, grain boundary engineering, mechanical properties, pressureless sintering, reinforcing additives, SiC, sintering techniques, spark plasma sintering

## Abstract

Silicon carbide (SiC) ceramics are among the most attractive materials for lightweight armor because they combine low density (3.0–3.2 g/cm^3^), high hardness, and high thermal and chemical stability; however, their densification remains challenging because of strong covalent bonding and low self-diffusion. This review analyzes the main sintering routes used for armor-grade SiC ceramics, including solid-state sintering, liquid-phase sintering, hot pressing, gas-pressure sintering, hot-isostatic pressing, ultra-high-pressure sintering, two-step sintering, and spark plasma sintering, together with additive systems based on B, C, Al_2_O_3_, Y_2_O_3_, MgO, CaO, and rare-earth oxides. Reported data show that solid-state sintering typically requires 2100–2300 °C and yields 90–95% relative density, whereas hot pressing and liquid-phase sintering achieve 96–99% density at lower temperatures, generally with a flexural strength of 350–800 MPa, fracture toughness of 3.5–7.0 MPa·m^1/2^, and hardness of 20–30 GPa. Among the reviewed methods, spark plasma sintering provides near-theoretical density (≥99%) together with the most favorable combination of strength (up to 850 MPa) and hardness (up to 35 GPa). Overall, liquid-phase sintering and spark plasma sintering offer the most favorable balance between densification, microstructural control, and armor-relevant mechanical performance.

## 1. Introduction

**Figure 1:**
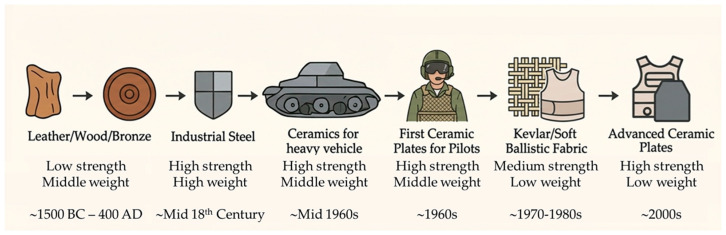
Historical development of armor materials.

Throughout history, the quest for effective armor materials has been driven by the need to balance protection, weight, and durability. Early armor primarily utilized leather, wood, and then metals such as bronze and iron, which offered basic defense but were limited by their strength and weight [1]. The emergence of steel plates during the Industrial Revolution significantly advanced ballistic protection due to steel’s superior hardness, toughness, and manufacturability (Figure 1).

Ceramic materials were first used as armor for heavy vehicles in World War I, and the first ceramic composite armor was a body armor insert plate, otherwise known as a hard armor plate, used in 1965 to provide lightweight protection against high-velocity projectiles [2].

**Figure 2:**
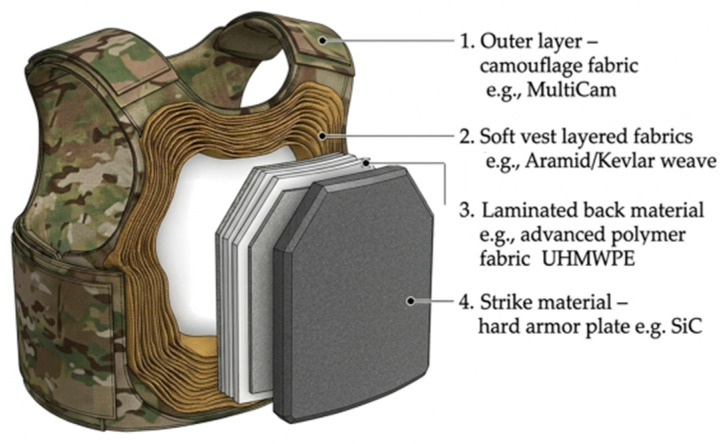
Typical layers of ceramic armor for personnel.

Over the past decades, significant research has focused on developing lightweight armor systems for personnel, vehicles, and structures, emphasizing their ballistic performance. Advanced ceramics play a key role in these systems by dissipating projectile energy through fracture upon impact, unlike metals, which rely on plastic deformation. Typical ceramic armor consists of monolithic or composite ceramic–metal plates backed by high-strength fibers or polyethylene layers (Figure 2). Upon high-velocity impact, the ceramic shatters, and the residual energy is absorbed by the soft reinforced backing material [3,4,5].

In the field of ceramic armor, research shifted toward lightweight, high-hardness materials capable of absorbing and dissipating impact energy. Traditional ceramics such as alumina (Al_2_O_3_) and silicate-based ceramics were early candidates, but their brittleness posed limitations [4,5,6,7]. Advances in materials science led to the development of ultra-hard ceramics like silicon carbide (SiC) and boron carbide (B_4_C).

Among advanced ceramic materials, silicon carbide (SiC) has emerged as one of the most technologically important ceramics due to its exceptional combination of physical, mechanical, and chemical properties. Owing to its relatively low cost and outstanding characteristics, including high corrosion resistance, superior hardness, high modulus of rupture, high thermal conductivity, low density, and excellent durability, SiC has attracted significant scientific and industrial interest [7,8,9,10,11]. As a result, SiC ceramics have found widespread applications in modern technologies, particularly in the defense, machine building, energy, and advanced manufacturing sectors.

Understanding and controlling the sintering behavior of SiC is essential for optimizing its mechanical properties, its microstructure, and hence, its effectiveness as a ballistic armor material. Recent research focuses on sintering methods such as hot pressing (HP), spark plasma sintering (SPS), and pressure-assisted processes to produce dense, defect-free SiC ceramics for advanced protection systems.

With regard to defense applications, silicon carbide (SiC) is one of the most widely used ceramic materials for armor systems. In recent years, significant attention has been directed toward the development of advanced protective elements, the effectiveness of which is largely determined by surface density, defined as the mass per unit area; lower surface density generally corresponds to improved ballistic performance [7]. Advances in the production of high-hardness ceramics and high-strength fibers have enabled the development of new types of armor systems capable of effectively dissipating projectile energy and significantly altering the impact dynamics on the protected structure [3,4,5,7]. For example, the combination of hard ceramics and high-strength polymer composite in a protective composition ensures the complete or partial destruction of the bullet, the residual energy of which is then absorbed by the composite substrate and other elements included in the protective composition [7,12]. Throughout the years, the main ceramic material for different armor was Al_2_O_3_ ceramics, but in the last years, a significant increase in the share of vests using SiC ceramics has been observed. The reason is the optimal intermediate position in properties and cost between, for example, B_4_C (more expensive) and Al_2_O_3_ (lower in required properties) [7].

A distinctive feature of SiC ceramic materials is the diversity of technological processes for obtaining products with various properties. On the other hand, there are some difficulties in the densification of SiC without grain growth or degradation of mechanical properties because of its strong covalent bonding nature and low self-diffusivity [8,10,13]. One way to densify SiC is sintering at very high temperatures under high pressures [8,9,13]. Another way, or additional way, to enhance the densification of SiC is the use of sintering additives [8,10,11,13,14,15,16,17,18].

For the correct selection of a sintering additive, it is important to consider the reaction between SiC and the sintering additives. The sintering additives react not only with SiC but also with silicon dioxide (SiO_2_), which is usually present on the SiC powder surface. These reactions form a wide range of phases, and the reactivity of SiC with the sintering additives is not fully understood. So, due to the limitations of theoretical understanding, attempts have been made to identify efficient sintering additives for SiC through the calculation of Gibbs free energy along with trial and error [13,14,15,16,17,18,19,20,21,22,23,24].

Also, based on theoretical calculations and the physical and chemical properties of SiC and additives, it is important to select a sintering technique that ensures the production of the required ceramic materials with the desired properties. Recent developments focus on optimizing the sintering parameters and additive systems to tailor the microstructure and phase composition, thereby achieving SiC ceramics with superior mechanical properties such as high flexural strength (>400 MPa), hardness (>25 GPa), and improved fracture toughness (>4 MPa·m^1/2^), suitable for high-performance armor systems under extreme service conditions [8,9,10,11,13,25,26,27,28,29,30]. With the above background, this review concerns sintering SiC ceramics with enhanced mechanical properties suitable for armor applications.

## 2. Mechanical Properties of SiC Ceramics

A number of criteria must be considered when selecting materials for use in an armor system for protection against ballistic threats. These include the nature of the anticipated threats, constraints on volume and weight, and overall system cost [5,6,7,31,32]. Due to the diversity of these requirements, no single armor material is optimal for all use cases.

For defeating higher-level threats, ceramic or metallic inserts are often necessary. Ceramics with low densities (2.5–4.0 g/cm^3^) offer significant weight advantages compared to traditional armor steels (density ~7.8 g/cm^3^) while maintaining higher hardness at equivalent thicknesses [5]. Among the ceramics with a low density and increased properties of hardness and armor resistance, hot-pressed B_4_C is favored, but the high cost, especially in mass production management, makes it necessary to give preference to other materials, of which Al_2_O_3_ is acceptable, but relatively lacking in armor properties. The disadvantages of both materials are leveled in SiC, which, in terms of its properties and cost, occupies an intermediate position between B_4_C and Al_2_O_3_. Table 1 shows some examples of commercially available SiC ceramics used for armor applications with important properties that serve in resisting ballistic impact.

Another reason could be that despite B_4_C’s superior hardness compared to SiC, B_4_C has low resistance against extra tenacious threats, due to shear localization leading to amorphization under high-pressure impact conditions [33,34,35,36]. SiC is a line compound and does not undergo amorphization under high applied dynamic pressures, consequently becoming a ceramic material of choice against more tenacious projectiles [34,35].

Due to the extremely short timescales involved in ballistic events (nanoseconds), no single material property has been consistently linked to armor performance [3,4,5,6,7,37]. Therefore, empirical ballistic testing remains essential to validate the effectiveness of any armor configuration against specific threats [6,7,37]. However, several fundamental material properties are routinely used for preliminary screening of ceramics for armor applications. These include the following [6,7,38]:

- Density: Influences the overall weight of the armor system (desired to be as low as possible, 3.0–3.2 g/cm^3^ for personal armor and 3.5–4.0 g/cm^3^ for vehicle armor).
- Hardness: Contributes to damaging the incoming projectile. Typically requires at least 20 GPa, depending on the specific level and intended application (e.g., personal body armor or vehicle armor).
- Fracture Toughness: Critical for resistance to cracking and multi-hit durability. Desirable range is at least 3–5 MPa·m^1/2^ for basic armor, and more than 5–6 MPa·m^1/2^ preferred for high-performance applications.
- Strength: Important for withstanding impact and multi-hit resistance. Usually, should be higher than 400–500 MPa for advance armor ceramics.
- Elastic Modulus: Related to stiffness and wave propagation. Desirable range is at least 300–400 GPa.

In general, the ceramic’s hardness should exceed that of the projectile to maximize its protective effect. High fracture toughness is also essential, as it reduces the likelihood of brittle fracture and improves the ability to resist multiple impacts [39]. However, an inverse relationship often exists between hardness and toughness—materials with extremely high hardness tend to have lower toughness and vice versa. Thus, selecting ceramics for specific threats involves balancing these competing properties. Typically, higher-hardness ceramics are chosen to protect against tool steel and tungsten carbide projectiles [7,31].

Additives along with porosity and grain size are microstructural aspects that affect all properties. Eutectic additives contribute to an increased hardness and elastic modulus, as well as flexural strength and fracture toughness, improving the ceramic’s resistance to projectile penetration. In ceramics like SiC, porosity is often considered a minor phase and can arise from different sintering techniques—such as HP, LPS or SSS, where the last may also include graphite or carbon in the product [6,40,41]. While these methods typically leave some residual porosity, reaction-bonded SiC generally lacks porosity but contains free silicon as a minor phase. These minor phases negatively impact key properties critical to ballistic performance [6,32].

Mechanical properties are also affected by the microstructure scale or the grain size. In general, both hardness and strength tend to decrease as the grain size increases [42,43]. Therefore, precise control of microstructural features is essential to optimize the ceramic performance, particularly for ballistic applications [6,32].

**Figure 3:**
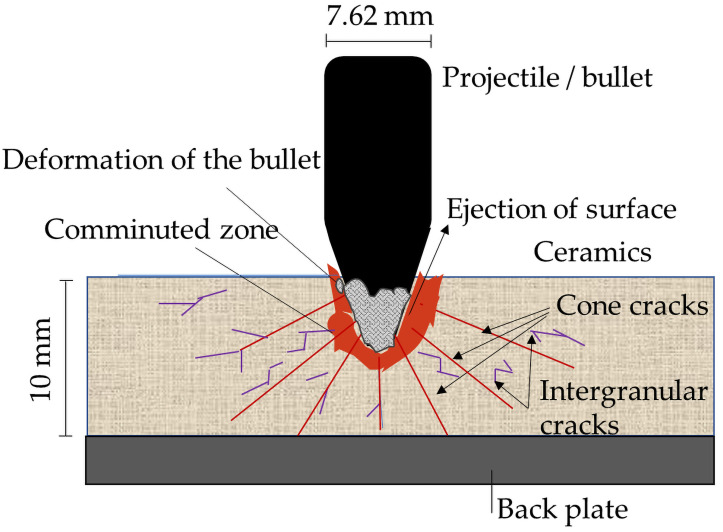
Deformation of the bullet and ceramic tile during ballistic impact.

During ballistic impact, extremely high strain rates occur at the point of contact, while strain rates decrease with the distance from the impact site (Figure 3). In these lower-strain regions, quasi-static fracture behavior becomes relevant and can provide insight into failure mechanisms [3,4,5,6,7,12]. Generally, materials exhibit more brittle behavior at higher strain rates.

SSS SiC typically fractures in a transgranular manner, leading to lower fracture toughness. Hot-pressed and liquid-phase sintered SiC shows predominantly intergranular fracture, sometimes with a transgranular component, resulting in higher fracture toughness [6,20,44,45,46].

Intergranular fractures follow complex paths along grain boundaries, requiring more energy and creating a larger fracture surface area, which enhances toughness [44,45,46,47]. However, this comes at the cost of hardness: for example, liquid-phase sintered SiC has lower hardness compared to SSS SiC, reflecting the typical trade-off between hardness and fracture toughness [6,39,47].

## 3. SiC Ceramic Sintering Aspects

The development of theoretical foundations for understanding the sintering process is based mainly on the advances in the sciences of the condensed state of matter—solid state physics and chemistry, crystal physics and crystal chemistry, and the theory of liquid structure. Various concepts of the kinetics of sintering processes have been developed to date, based both on theoretical consideration of particle interactions and on empirical processing of experimental sintering data. When considering the mechanisms and classification of sintering processes, the state of the phases involved in mass transfer is primarily taken into account. The two main groups of processes are divided into solid-phase and liquid-phase sintering. When classifying sintering processes, it is also common to distinguish between HP and reaction sintering.

The effective sintering of SiC materials includes various parameters that need to be taken into account. During the consolidation of SiC, a high sintering temperature is typically necessary, resulting in a material with enhanced grain growth. Thus, to minimize this grain growth, incorporating reinforcing phases has proven to be effective as it reduces the sintering temperature and enhances the overall properties of the ceramics. Nonetheless, certain reinforcing phases with characteristics inferior to those of the matrix are consistently seen to hinder the properties of the ceramic materials. On the other hand, some exhibit a rising shrinkage rate, resulting in greater densification at a reduced sintering temperature along with enhanced mechanical properties. Moreover, pressure can be applied to provide an extra driving force for consolidation, which reduces the sintering time and decreases the temperature. Different techniques with various reinforcement stages have been utilized for the consolidation of SiC, such as hot-pressing, pressureless sintering (PLS), hot isostatic pressing (HIP), microwave sintering, SPS, and others.

The primary factors influencing the sinterability and resulting microstructure of a powder compact can be classified into two main categories: material-related variables and process-related variables. Material variables pertain to the raw materials and include the chemical composition of the powder, particle size and shape, size distribution, and degree of agglomeration. These characteristics directly affect the compressibility and sinterability (i.e., densification and grain growth) of the powder. For powder mixtures containing multiple components, the homogeneity of the blend is critical. To enhance homogeneity, both mechanical methods (e.g., milling) and chemical techniques (e.g., sol–gel and coprecipitation) are commonly employed. Process variables are primarily thermodynamic and include the sintering temperature, duration, atmosphere, pressure, and heating/cooling rates. While many studies have explored the effects of temperature and time, the influence of the atmosphere and pressure is often more complex and significant in practical applications.

According to the literature [6,8,41,42,43,44,48], factors such as the powder particle size, agglomeration, and green body formation stage are especially important across all types of sintering processes. Fine powders with high surface areas promote sintering at lower temperatures due to their higher reactivity. However, nanopowders tend to exhibit poor rheological properties, such as electrostatic behavior and agglomeration, making handling and shaping challenging. Rheology plays a vital role by enabling uniform die filling and influencing densification through porosity elimination during sintering.

To process fine powders effectively, optimizing the pre-compaction step—using methods like slip or tape casting—is essential. These casting methods simplify powder handling but require the addition of organic binders, which necessitates an additional pre-sintering step to remove residues. Alternatively, pressure-assisted sintering techniques, such as HP or SPS, allow direct powder compaction and sintering in a die, where uniform filling is key to achieving a consistent final product. For example, Lara et al. [49] used SPS to sinter commercial and additive-free β-SiC powder, achieving 98% density at 2100 °C under 150 MPa. Boehmler et al. [50] found that even when the same initial powder is used, its conditioning greatly impacts the resulting ceramic. Granulated SiC powder showed the best compaction and produced the densest ceramics with the finest grains.

To achieve higher fracture toughness, nanopowders are usually used because they have greater specific surface activities and surface areas in comparison with micron powders by enhancing the sinterability [43,51,52]. However, simply reducing the grain size does not always result in higher density or improved mechanical properties. Lorrette et al. [52] found that SiC ceramics sintered with 80 nm powders showed slightly better mechanical properties than those sintered with 30 nm powders.

The choice between α-SiC and β-SiC also influences processing outcomes. β-SiC is generally preferred for producing dense, high-strength ceramics due to its lower sintering temperature and minimal pressure requirements. β-SiC is in a metastable phase at room temperature, which transforms to polytypic forms of the α-SiC phase at elevated temperatures, with the 2H polytype becoming unstable over 1600 °C and converting into different polytypes depending on the temperature. Above 2000 °C, β-SiC will not exist unless there are some aids that stabilize and increase the transformation temperature [53]. In contrast, α-SiC is better suited to applications where grain growth and thermal stability are needed, such as in HP or reaction bonding processes.

Seeding the β-SiC-based initial powder mixtures with large α-SiC or β-SiC grains helps control and facilitate the transformation during heat treatment, resulting in the in situ reinforcement with elongated grains, and reduces weight loss and microstructural degradation caused by prolonged heat treatment [54,55,56,57,58]. Kim et al. [56] indicated that both α-SiC and β-SiC can be used to optimize the microstructure and, consequently, the mechanical properties of SiC ceramics.

Kim et al. [58] studied how varying the ratio of α-SiC to β-SiC in starting powder mixtures—while maintaining similar particle sizes—affects the microstructure and mechanical properties of liquid-phase sintered SiC-based ceramics. Their findings indicate that there is an optimal proportion of α-SiC that maximizes both flexural strength and fracture toughness. Generally, increasing the amount of α-SiC enhances flexural strength, but it tends to reduce fracture toughness. These mechanical property changes were closely linked to alterations in the material’s microstructure. A higher α-SiC content promoted a finer, more uniform microstructure with equiaxed grains. However, the best combination of microstructural refinement and mechanical performance was observed when the α-SiC content did not exceed 10%. At this 10% level, the material displayed the highest aspect ratio of elongated grains while still maintaining a refined grain structure. Moreover, the presence of α-SiC also contributed moderately to improved densification.

Powders used for sintering may have different impurities, which can significantly degrade the physicochemical properties of SiC ceramics. For instance, Yasar et al. [59] showed that a high oxygen content can lead to excessive grain growth and reduced hardness. Feng et al. [41] observed that impurities react with carbon additives to form gases during sintering, causing porosity and coarse grains, which negatively impact mechanical performance. Removing such impurities has been shown to greatly enhance the final mechanical properties.

**Figure 4:**
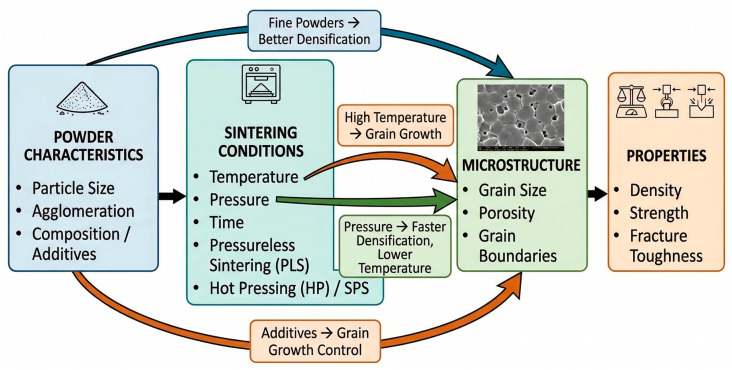
Schematic representation of relationships during SiC sintering.

The relationships between powder characteristics, sintering conditions, microstructure, and resulting properties of SiC ceramics are schematically illustrated in Figure 4. As shown, both material- and process-related parameters jointly control densification behavior and grain evolution, ultimately determining the mechanical performance of the final ceramic.

Examples of the application of various sintering techniques, variations in the parameters during sintering (temperature, pressure, time, heating rate), as well as the use of various additives are described in more detail in the following sections.

### 3.1. Solid-State Sintering of SiC Ceramics

Generally, SiC sintering can be divided into two categories based on the sintering additive’s mechanisms: liquid-phase sintering (LPS) and solid-state sintering (SSS). One method of densification that does not require the presence of liquid in the system is SSS.

Due to the highly covalent bond characteristics, relatively high temperatures are necessary for SSS of SiC in order to induce material densification and diffusion via several mechanisms. Diffusion, on the other hand, is the matter transport mechanism that encourages both densification and grain growth. Thus, appropriate settings of sintering that permit densification without concurrently promoting grain development are required for the production of highly dense ceramics with nanometric grains [23,24,45,60,61].

Three overlapping steps are often used in SSS. The first stage is distinguished by the development of necks between particles, which contributes little more than 2–3% to compact shrinkage, increasing contact between particles and raising the relative density to about 50–60%, with the first signs of grain growth appearing at the end [19,62]. During the intermediate stage, pores are intersected by grain boundaries due to equilibrium dihedral angles, and open pores begin to shrink, pushing the relative density close to 90% [22,62]. In the final stage, isolated closed pores form near the junctions of four grains, adopting nearly spherical shapes for energetic stability. Under steady conditions, these pores can shrink completely, marking the end of the sintering process.

The main mechanisms that contribute to neck growth and densification in polycrystalline materials are surface diffusion, lattice diffusion, vapor transport, grain boundary diffusion, and lattice diffusion along grain boundaries and dislocations [22,62,63]. The latter two promote densification, while the others mainly cause coarsening by enlarging necks without decreasing the distance between particle centers.

A fundamental prerequisite for sintering is that the grain boundary energy must be lower than approximately twice the solid/vapor surface energy [23]. Only under this condition can mass transport effectively reduce the system’s excess energy via the transformation of surface area into grain boundary area, followed by grain boundary migration and pore shrinkage [45].

In covalently bonded ceramics such as SiC, the grain boundary energy is sufficiently high to significantly inhibit pore shrinkage and densification [45,64]. Consequently, diffusion-controlled solid-state densification is virtually unattainable for pure SiC. Instead, grain growth predominates during sintering, presenting a kinetic limitation to achieving full densification. Due to its fully crystalline nature and well-defined grain boundaries, monolithic SiC typically exhibits transgranular fracture, resulting in low fracture toughness (~2–2.5 MPa·m^1/2^). This mechanical limitation restricts its utility in structural applications such as armor. To address this, sintering is commonly carried out in the presence of minor additions of densification aids, referred to as sintering additives or activators. The most widely used additive in SSS is elemental boron [20,24,46,65], which, however, is effective only in conjunction with carbon [45,66,67]. In fact, various boron-containing compounds have demonstrated enhanced mechanical performance in comparison with elemental boron [41,68,69,70].

Although the addition of B, C, or boron compounds has enabled successful sintering of SiC, the precise energetic mechanisms by which these additives promote densification are not yet fully understood [23,24,71]. The primary role of carbon is associated with the removal of the native SiO_2_ layer present on SiC particles, as well as the chemical bonding of gaseous by-products generated during SiO_2_ reduction.

Furthermore, aluminum (Al) and Al-containing compounds [72,73] have also been employed as sintering additives. In some cases, these additives facilitate densification via transient liquid-phase sintering or activated sintering pathways, rather than through conventional solid-state diffusion mechanisms.

The composition and thermal treatment of SiC with Al, B and C additions have a substantial impact on its mechanical properties, especially its fracture toughness. The intergranular glassy film’s degree of crystallinity and grain-aspect ratio are the main determinants of fracture toughness because intergranular fracture caused by the amorphous layer’s chemically weak surfaces predominates in this system. The material cracks in an intergranular cracking mode instead of a deadly transgranular cracking mode because the amorphous intergranular glassy film provides easy pathways for crack penetration. The bridging effect from the grains and zigzag crack penetration also greatly increase the fracture toughness. Grain morphology altered from elongated to bimodal to equiaxed as Al addition increased, while the grain-boundary film’s nature changed from amorphous to partially crystalline to entirely crystalline [8,45].

**Figure 5:**
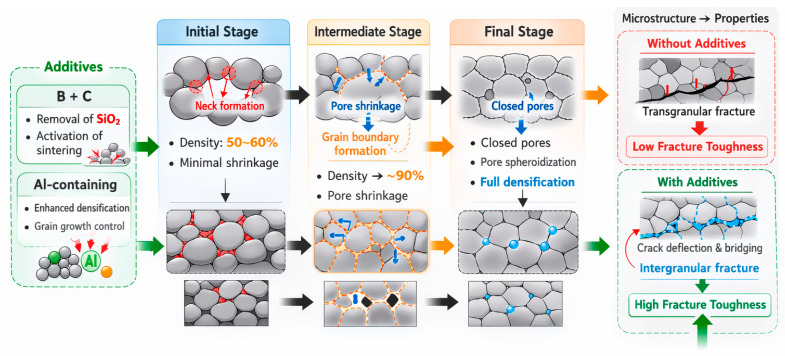
The role of additives in the process of solid-state sintering (SSS) of SiC ceramics.

Thus, solid-state sintering of SiC ceramics proceeds through a sequence of microstructural transformations, including neck formation, pore shrinkage, and final densification, as illustrated in Figure 5. The incorporation of sintering additives such as B, C, and Al plays a critical role in enhancing densification, modifying grain boundary characteristics, and improving fracture behavior, thereby significantly increasing the fracture toughness of the material.

### 3.2. Liquid-Phase Sintering of SiC Ceramics

Liquid-phase sintering (LPS) is a powder consolidation technique applied to multi-component systems at temperatures exceeding the solidus of at least one constituent, leading to the formation of a transient liquid phase during sintering [74]. This liquid can originate from the melting of a single component, eutectic reactions between multiple components, or heating an alloy to a temperature range between its solidus and liquidus, which promotes mass transport through highly diffusive liquid pathways. Compared to SSS, LPS operates at relatively lower temperatures, making it advantageous for industrial processing. However, the presence of liquid complicates the analysis of sintering mechanisms compared to solid-state processes.

The presence of a liquid phase significantly enhances mass transport, resulting in rapid microstructural evolution. Similar to SSS, a characteristic densification curve is observed over time. Notably, some densification already occurs in the solid state before reaching the LPS temperature, making the initial microstructure from SSS crucial to the process. When conventional heating is applied, densification levels exceeding 90% relative density can be achieved once sintering is activated. As the powder compact is heated, liquid forms and is drawn into finer capillaries due to differences in capillary pressure between small and large pores, causing particle rearrangement—an essential feature of LPS behavior [74].

Effective LPS is governed by several critical factors, including low contact and dihedral angles, sufficient volume of the liquid phase, high solubility of solids in the liquid, uniform packing of particles, homogeneous distribution of the liquid, and fine particle sizes. These parameters collectively enhance mass transport and microstructural uniformity during sintering [75].

The conventional model of LPS can be divided into three overlapping stages, as described in the literature [74,75,76]. The initial stage involves particle rearrangement, which is initiated as the temperature rises and a liquid phase begins to form. Capillary forces drive this rearrangement, redistributing both the liquid and the solid particles, particularly around fine pores. This process continues until a relatively stable packing configuration is achieved, which corresponds to a reduction in the densification rate over time. The extent of densification in this stage is strongly dependent on the quantity of liquid present—if sufficient, the rearrangement alone can lead to near-full densification of the ceramic body. The second stage is characterized by solution–precipitation mechanisms, where solubility and diffusivity within the liquid phase dominate the microstructural evolution. In this stage, finer grains dissolve into the liquid and subsequently reprecipitate onto larger grains, driven by differences in chemical potential. A key densification mechanism at this stage is contact flattening, where particle interfaces become more planar, enhancing densification while limiting grain growth. The final stage is primarily governed by SSS. At this point, the densification rate becomes significantly slower due to the formation of a rigid solid skeleton that impedes further particle rearrangement. Any ongoing densification occurs primarily through grain boundary diffusion mechanisms [74,75,76].

LPS proceeds through distinct stages influenced by time and temperature, including liquid formation, particle wetting and rearrangement via capillary forces, and solution–reprecipitation [75,77]. Among these, the stages involving liquid formation and particle rearrangement progress more rapidly than solution–reprecipitation. Additionally, SSS may initiate before the liquid phase emerges—typically at lower temperatures and over shorter durations—resulting in minor shrinkage of the compact. This early densification is mainly driven by mechanisms such as evaporation–condensation and surface diffusion [75].

Understanding the interplay between heating rate and sintering mechanisms is crucial for optimizing densification and microstructural control in LPS processes. The effect of the heating rate on the shrinkage and microstructure of LPS SiC ceramics was described by Ribeiro et al. [10,78], and despite an ongoing search, the general observations are well-supported: prior to liquid formation, slower heating increases shrinkage; at a given temperature, faster heating raises the peak shrinkage rate, enhancing densification via particle rearrangement; the shrinkage peak marks the breakdown of solid–solid bonds by the liquid; this peak intensifies with faster heating, indicating greater densification from rearrangement [78].

The liquid volume fraction is another important factor in LPS. For high-performance ceramic components, the liquid-phase content is typically kept below 5 vol% to prevent degradation of SiC properties [75]. However, in certain cases, a liquid content exceeding 25 vol% is necessary to effectively fill the gaps between solid particles and facilitate the sintering process [8].

In general, compared with SSS, LPS allows easy control of the microstructure and a reduction in processing cost, but degrades some important properties, for example, mechanical properties. One of the densification routes is using effective eutectic additives. One of the issues with using sintering aids for SiC is their active internal chemical interaction at the sintering temperatures. A variety of reactions results in gaseous species formation, thus leading to severe weight loss and an overall shift of the composition during heat treatment. Commonly, Al_2_O_3_, Y_2_O_3_, a combination of the latter, and a limited number of rare-earth oxides, normally in combination with Al_2_O_3_, have been reported to be effective sintering aids for SiC [11,29,58,79,80,81,82,83,84,85,86,87,88,89].

One of the foundational studies on PLS LPS of SiC using oxide additives was conducted by Liu et al. [90]. They used Al_2_O_3_ and Y_2_O_3_ introduced through reactions involving Al(OH)_3_ and HCl, and Y(OH)_3_ and HCOOH, respectively. Their research showed that adding either Al_2_O_3_ or Y_2_O_3_ alone did not significantly improve densification during PLS sintering at 2150 °C. However, the combined use of both additives increased the β-SiC density to 96.8%.

In order to lower sintering temperatures, pressure-assisted methods such as HP are commonly used in combination with LPS. According to Rahaman [75], pressure raises the chemical potential at particle contact points, which in turn enhance atomic diffusion. Can et al. [91], while studying different densification routes of SiC with Al_2_O_3_–Y_2_O_3_ additions, found that HP (1975 °C, 30 min, 30 MPa) resulted in 99% densities, and gas pressure (GP) sintering (1925 °C, 60 min, 8 MPa) produced densities from 90.5% to 98.8%. The difference in densification was attributed to evaporation rates.

**Figure 6:**
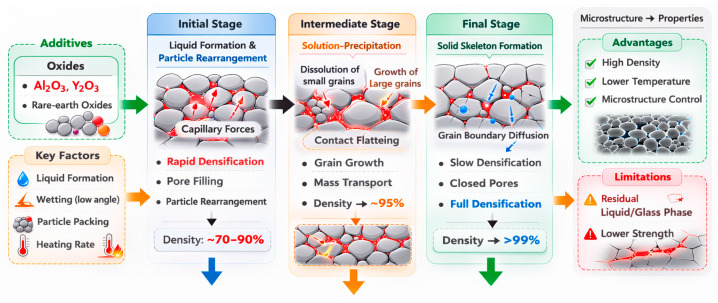
Additive effect on liquid-phase sintering of SiC ceramics.

A summarizing scheme is presented in Figure 6.

### 3.3. SiC Sintering Techniques

#### 3.3.1. Pressureless Sintering

**Figure 7:**
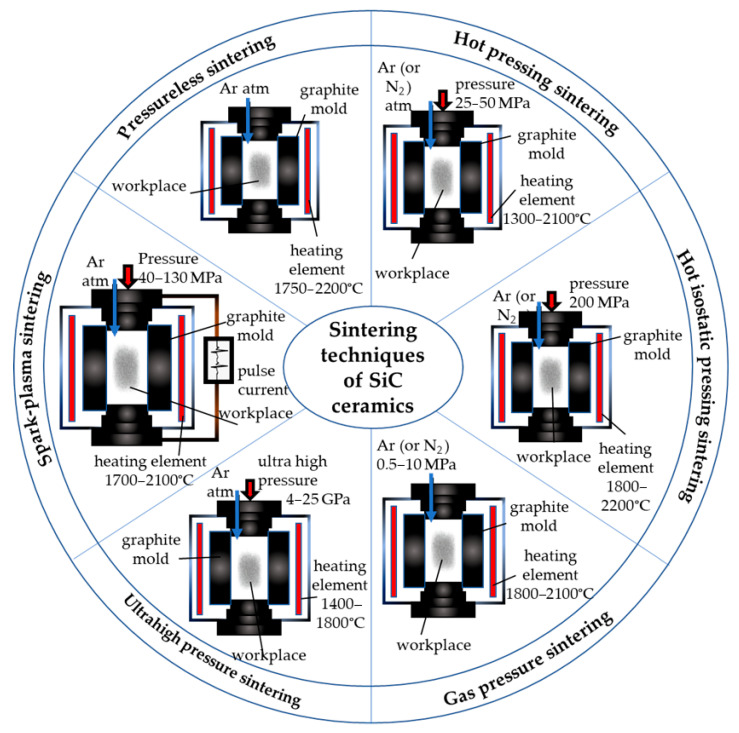
Typical sintering techniques of SiC ceramics.

Pressureless sintering (PLS) is the modest technique capable of densifying samples of any form and size without external pressure (Figure 7). The densification of pure SiC during PLS sintering is intrinsically difficult due to the predominance of the evaporation–condensation mass transport mechanism at high temperatures. The relatively high vapor pressure of Si-containing species leads to material evaporation from smaller particles and condensation on larger grains, which promotes grain coarsening rather than densification. Consequently, significant shrinkage is limited in additive-free systems. The incorporation of boron and carbon additives is therefore commonly employed to suppress this effect and activate diffusion-controlled sintering mechanisms, facilitating densification of SiC ceramics.

In contrast, oxide additives such as Al_2_O_3_, Y_2_O_3_, MgO, and rare-earth oxides promote densification of SiC through liquid-phase sintering. These additives react with the native SiO_2_ layer present on the surface of SiC particles at elevated temperatures, forming a transient liquid phase that enhances particle rearrangement and mass transport. Densification then proceeds predominantly via solution–precipitation mechanisms within the liquid phase, which facilitates pore elimination and suppresses grain coarsening associated with the evaporation–condensation mechanism. As a result, oxide-assisted sintering enables significantly higher densification of SiC ceramics at lower temperatures compared with additive-free systems.

There are some research studies on PLS-sintered SiC ceramics that assessed the fracture toughness, or flexural strength, as well as the sintered density. She et al. [79] investigated a SiC ceramic with 5 to 20 wt% Y_2_O_3_ and Al_2_O_3_ with different ratios at 1850–2000 °C for 1 h in an Ar atmosphere without an applied pressure, resulting in characteristics such as a fracture toughness, flexural strength and hardness of the ceramic of 6.3–7.5 MPa·m^1/2^, 480–650 MPa and 17–20 GPa, respectively.

Kim and Kim [11] created a SiC ceramic with 5.02 wt% Al_2_O_3_ + 2.78 wt% Y_2_O_3_ + 0.71 wt% MgO + 0.49 wt% CaO additions at low temperatures (1750–1850 °C) over 2 h in an argon atmosphere without applied pressure. The ceramic’s fracture toughness, flexural strength and hardness were 4.8–5.6 MPa·m^1/2^, 220–347 MPa and 23–29 GPa, respectively.

Further, a SiC ceramic was created by Malik et al. [28] using 2.36 wt% SiO_2_ + 5.48 wt% Al_2_O_3_ + 3.03 wt% Y_2_O_3_ + 0.74 wt% CaCO_3_ additives at 1850 °C for 2 h in an Ar environment without any pressure applied. The ceramic’s flexural strength was 686 MPa, hardness 26 GPa and fracture toughness 5.4 MPa·m^1/2^.

#### 3.3.2. Hot Pressing

The mechanical pressure applied in HP boosts the force for densification by helping the rearrangement of particles during the initial phase of sintering (Figure 7). Once a relatively high density is attained, the mechanical pressure counteracts the internal pore pressure, decreasing the porosity, without elevating the driving force for coarsening. The duration and temperature of sintering can be greatly minimized when HP is applied together with an LPS additive, leading to enhanced mechanical properties.

Works on HP of SiC, obtaining materials with enhanced mechanical properties, have been reported by many researchers [42,89,91,92,93,94,95,96,97,98]. Li et al. [98] mixed SiC and TiN powders with boron, and HP the mixture at 2000 °C in an argon atmosphere, obtaining materials with 80% increased fracture toughness with 20 vol% of TiB_2_ added. Hao et al. [99] fabricated SiC ceramics with 0.5 wt% B_4_C and 0.5 wt% C as sintering additives by HP at 2000 °C, reaching 25.9 GPa hardness. Chae et al. [93] prepared SiC compacts containing 30 vol% TiC, and various contents of Cr_3_C_2_, by hot-pressing at 1900–1950 °C, and obtained ceramics with a high flexural strength of 750–810 MPa along with a fracture toughness enhancement to 4.6 MPa·m^1/2^. Hong et al. [32] prepared SiC ceramics by HP (80 MPa, 1900 °C, 1 h, Ar atm.) with 8 wt.% Al_2_O_3_/Y_2_O_3_ (molar ratio 3/2) addition and studied the effect of the layer structure on the ballistic performance, obtaining ceramics with a fracture toughness of 6.8–7.4 MPa·m^1/2^.

#### 3.3.3. Gas Pressure Sintering

Gas pressure (GP) sintering: In the initial sintering phase, the sample is sintered until pores close, after which, in the second phase of sintering, internal pores reduce their size and eliminate under GP (Figure 7). The benefit of GP sintering is the variety of sample shapes that can be made. Biswas et al. [100] prepared 99.5% densified SiC by GPS with the addition of Gd_2_O_3_-Ho_2_O_3_, Dy_2_O_3_-Ho_2_O_3_, Y_2_O_3_-AlN, and Lu_2_O_3_-AlN, which improved fracture toughness up to 4.8 MPa·m^1/2^. Mandal et al. [101] also investigated the GPS of SiC at 1700—1950 °C under a N_2_ pressure of 6 bars with AlN additives and obtained ceramics with 21.26–25.36 GPa hardness. Izhevskyi et al. [43] carried out GP sintering of SiC at 1950 °C under Ar pressure up to 2 MPa with the addition of 10.65 wt% Y_2_O_3_ + 2.9 wt% AlN. Fracture toughness of prepared ceramics improved from 3.6–3.8 to 4.7–5.9 MPa·m^1/2^ with the duration of heat treatment 1–16 h while hardness decreased from 18.5–22.5 to 17.5–21 GPa.

#### 3.3.4. Hot-Isostatic Pressing

Hot-isostatic pressing (HIP) is the use of isostatic GP at several MP, where a green body is firmly encased in a glass or metal container and sealed under vacuum (Figure 7). As the sample is heated to the sintering temperature, the closed porosity is collapsed by gradually increasing the GP during heating to as high as 200 MPa [102].

Jihong et al. [103] described the effect of additives, such as B_4_C, AlN and Al_2_O_3_, on the sintering of α-SiC by HIP at 1850 °C by applying 200 MPa for 1 h. HIPed, Al_2_O_3_-doped SiC had an ultrafine-grained microstructure with a grain size varying from 0.5 to 2.5 μm, and showed a flexural strength above 570 MPa and a fracture toughness higher than 5.17 MPa·m^1/2^. Shaoming et al. [104] investigated the effect of the additive-phase TiC particle size on the properties of SiC by HIP at 1850 °C and 200 MPa, and reported that a composite with coarser TiC particles (1 μm) shows higher fracture toughness, while they noted the similarity of the bending strength and lower Vickers hardness compared with the composite with finer TiC particles (0.1 μm). Tani [105] achieved a preparation of SiC ceramics with a fracture toughness from 4.7 to 6.2 MPa·m^1/2^, and flexural strengths from 690 to 826 MPa, by varying the doped carbon source and temperature (2000–2200 °C) in HIP, applying 200 MPa.

#### 3.3.5. Ultra-High-Pressure Sintering

Ultra-high-pressure sintering (UHPS) is performed by applying pressures up to several GPa in order to reduce the sintering times (Figure 7). Gadzira et al. [106] investigated UHPS at 1400–1800 °C under 4–6 GPa for 1 min to sinter SiC-C and form highly hard ceramics up to 29.0–39.5 GPa. Although mechanical properties were not highlighted, there are a few reports on the densification of SiC by UHP [107,108]. Recently, high-purity SiC (average 55 nm size) applied in HF acid treatment to eliminate surface SiO_2_ was used in order to obtain highly hard (31.9–34.8 GPa) SiC ceramics by UHPS at 1400–1500 °C under 5–25 GPa [9].

#### 3.3.6. Two-Step Sintering

The two-step sintering technique involves sintering with thermal pretreatment at a low sintering temperature, followed by a second stage at an elevated temperature, or vice versa.

Chu et al. [109] introduced the two-step sintering (TSS) technique, and later, Chen and Wang [110] proposed a modification, which become commonly used when speaking of two-step sintering. This method involves inhibiting the rapid grain growth that typically takes place in the final sintering step. Heating at a high temperature is performed, and then the structure is frozen. While rapid cooling at a steady pace inhibits grain growth, densification is permitted. Numerous ceramic materials can benefit from the technique’s effective use, which allows for high-density microstructure refinement and enhances the materials’ various qualities [61].

Cho et al. [92] described the composites fabricated from β-SiC and TiB_2_ powders with the liquid forming additives of Al_2_O_3_ and Y_2_O_3_ by hot-pressing at 1850 °C and following annealing at 1950 °C without pressure to enhance the grain growth. Obtained ceramics showed an increase in fracture toughness from 4.4 to 6.7 MPa·m^1/2^ and a slight decrease in flexural strength from 571 to 550 MPa.

Magnani et al. [111] used α-SiC with 0.5 wt% boron and 3.0 wt% carbon, to produce SiC ceramics by PLS sintering, comparing conventional sintering at 2200 °C for 1 h in an Ar stream and the two-step sintering method with T1 at 2100 °C and T2 at 2050 °C for 7 h. There was sharp grain growth (more than 100 μm) in conventional sintering, whereas the grain size was approximately 30 μm in two-step sintering. While the relative density, hardness and fracture toughness values were similar, flexural strength was higher (341 MPa to 556 MPa) when the two-step sintering method was used.

#### 3.3.7. Spark Plasma Sintering

Ceramics may be sintered rapidly by SPS, which applies electric current pulses to the sample’s opposite ends inside a conductive die, so sintering occurs through an exothermic reaction within a powder (Figure 7). The key benefits of SPS are that it can improve the densification of ceramics that are difficult to sinter by delivering a direct or pulsed electric current and a uniaxial load to a powder compact at the shorter sintering time (within a few minutes). As a result, densification occurs before significant coarsening of SiC grains takes place. In addition, the rapid heating and pulsed direct current in SPS may influence grain boundary chemistry by inducing localized Joule heating and enhancing diffusion at particle contacts. These effects can promote the removal or redistribution of surface oxide films and accelerate mass transport along grain boundaries. Consequently, SPS enables rapid densification while maintaining a fine-grained microstructure, whereas conventional hot pressing, with slower heating rates and longer dwell times, often results in pronounced grain coarsening.

Tamari et al. [112] reported the SPS of SiC containing 5 wt% Al_2_O_3_ and 2 wt% Y_2_O_3_ under 30 MPa with a 5 min dwell at 1800 °C. From the obtained results, they concluded that rapid grain growth leads to lower strength; in this work, the strength of dense bodies sintered by SPS was higher (850 MPa at 1800 °C in 5 min) than that of those by hot-pressing (730 MPa at 2000 °C in 30 min).

Lomello et al. [113] densified SiC green bodies prepared via aqueous slip-casting to 95% using SPS. Varying the sintering temperatures (1700–1900 °C) affected microstructure development, particularly with ball milling, which activated particle surfaces and caused grain coarsening at higher temperatures. The resulting samples showed good mechanical properties: hardness of 25 GPa and toughness of 5.0 MPa·m^1/2^.

Hayun et al. [114] sintered SiC at 2050 °C for 10 min under 69 MPa and 400 °C/min heating. The fully dense SiC achieved excellent mechanical properties: 6. MPa·m^1/2^ fracture toughness, 440 GPa Young’s modulus, 490 MPa flexural strength, and 32 GPa hardness.

There are some reports on SPS regarding the addition of TiC or TiB_2_ to the sintered SiC bodies. Shinoda et al. [115] synthesized SiC–TiC ceramics using Al_2_O_3_ and TiO_2_ additives. When sintered at 1800 °C under 50 MPa, the samples do not show a high value of fracture toughness as a result of their ultrafine-grained microstructure. Luo et al. [116] fabricated SiC–TiC composites via SPS without additives. They achieved up to 98% density, with hardness and strength improving with a higher TiC content and sintering temperature. The 30% TiC composite showed the best mechanical performance (6.25 MPa·m^1/2^ fracture toughness and 28.1 GPa hardness).

Cho et al. [117] produced SiC–TiC composites (10–40 wt% TiC) with Al, B, and C additives through SPS. The 40 wt% TiC composite sintered at 1800 °C for 20 min, fully densified and reached 720 MPa strength and 6.3 MPa·m^1/2^ toughness. The researchers indicated that the strength increased with the TiC content, and the opposite occurred with a longer holding time.

#### 3.3.8. Comparative Analysis of Sintering Strategies for SiC Armor Ceramics

Following the general overview of silicon carbide as a promising material for lightweight armor systems, it is essential to compare the principal sintering strategies used to produce armor-grade SiC ceramics. Unlike conventional structural ceramics, SiC intended for ballistic protection must combine high hardness, sufficient fracture toughness, and controlled fracture behavior to ensure effective energy dissipation under high-strain-rate impact loading.

The sintering route plays a decisive role in defining these characteristics, as it governs densification kinetics, grain growth, and grain-boundary chemistry. Therefore, a comparative analysis of sintering strategies provides a necessary framework for understanding the structure–property relationships discussed in subsequent sections.

Conventional solid-state sintering of SiC typically requires temperatures exceeding 2100 °C due to the strong covalent bonding and low self-diffusion coefficients of SiC. As a result, this approach often leads to incomplete densification, residual porosity, and coarse-grained microstructures, which are generally unfavorable for armor applications. Consequently, solid-state sintered SiC is of limited relevance for modern ballistic protection systems.

Pressure-assisted and liquid-phase-assisted sintering routes enable densification at lower temperatures and provide improved control over microstructural evolution. In particular, liquid-phase sintering using oxide-based eutectic systems has been shown to significantly reduce the sintering temperature while maintaining high density and mechanical strength, making it attractive for armor ceramics [118]. Spark plasma sintering (SPS), characterized by rapid heating rates and short dwell times under applied pressure, further suppresses grain growth and enables the production of near-theoretical-density SiC ceramics with a fine and homogeneous microstructure [119].

Table 2 summarizes the key features of the main sintering strategies employed for SiC armor ceramics and highlights their relevance to ballistic applications.

From an armor perspective, liquid-phase sintering and SPS offer the most favorable balance between densification efficiency, microstructural control, and mechanical performance, whereas solid-state sintering is generally unsuitable for high-performance armor ceramics.

In armor-grade SiC ceramics, sintering additives should not be considered solely as densification aids. Their functional role extends to modifying the grain-boundary chemistry and fracture behavior, which are critical for impact resistance. Oxide-based additive systems such as Al_2_O_3_–Y_2_O_3_ and MnO–Al_2_O_3_–SiO_2_ form transient liquid phases during sintering, facilitating particle rearrangement and pore elimination while simultaneously influencing grain-boundary characteristics.

Ultrasound-assisted liquid-phase sintering with eutectic oxide additives enables a reduction in sintering temperature to approximately 1800 °C and results in dense SiC ceramics exhibiting a flexural strength of about 440 MPa and microhardness close to 30 GPa—values directly relevant to armor applications [118]. The use of oxide nano-additives in SPS further enhances sintering activity and suppresses grain growth, leading to increased hardness and strength while preserving a fine-grained microstructure [119].

A functional classification of sintering additives for armor-grade SiC, based on their dominant mechanisms of action, is presented in Table 3.

These observations highlight that additive selection for armor ceramics must prioritize not only densification efficiency but also the promotion of favorable fracture mechanisms under dynamic loading.

The fracture behavior of SiC ceramics under ballistic loading is strongly influenced by microstructural features established during sintering. Solid-state sintered SiC typically exhibits transgranular fracture, which limits its ability to dissipate impact energy. In contrast, SiC ceramics produced by liquid-phase sintering and SPS predominantly fracture along grain boundaries, promoting crack deflection and branching.

Microstructural studies of liquid-phase sintered SiC reveal equiaxed grains and modified grain-boundary phases that contribute to enhanced fracture toughness without a significant reduction in hardness [118]. Similarly, SPS-processed SiC ceramics with oxide nano-additives demonstrate a fine-grained and homogeneous microstructure associated with high strength and microhardness values reaching 35 GPa [119].

Table 4 summarizes qualitative relationships between the sintering route, fracture mode, and expected ballistic response based on trends widely reported in the literature. The values vary depending on the composition, additives, and microstructure, but consistent trends are established.

From the standpoint of ballistic protection, intergranular fracture mechanisms are advantageous, as they increase the fracture surface area and enhance energy absorption during impact.

In addition to mechanical performance, the selection of a sintering strategy for armor ceramics must consider energy consumption, processing time, and scalability. Traditional hot pressing remains a reference technology for high-performance armor ceramics but is associated with high energy costs and limited production efficiency.

Liquid-phase sintering represents a more scalable and industrially viable route, combining moderate processing temperatures with the ability to produce dense armor-grade SiC ceramics using conventional furnaces [118]. Spark plasma sintering, although currently limited in component size, offers unparalleled control over the microstructure and rapid consolidation, making it particularly attractive for advanced armor components and functionally graded materials [119].

Compared with conventional techniques, SPS provides enhanced control over microstructural evolution due to its extremely high heating rates and short dwell times. Rapid heating enables densification to occur before significant grain growth develops, resulting in a refined microstructure with limited grain coarsening. In addition, the pulsed electric current passing through the compact is believed to promote localized Joule heating at particle contacts, which enhances diffusion along grain boundaries and particle interfaces [112,113,114,115]. Several studies [112,113,114,115] suggest that this effect may facilitate the disruption or redistribution of surface oxide films present on SiC particles, thereby accelerating mass transport during sintering. As a result, SPS can achieve near-full densification while preserving a fine-grained microstructure, whereas the slower heating rates typical of conventional hot pressing allow more time for grain growth during densification.

In contrast, LPS relies on oxide additives that form a transient liquid phase during sintering. The presence of a liquid phase significantly enhances mass transport and enables near-full densification at relatively lower temperatures [28,29,76,77,78,118]. However, these additives often lead to the formation of intergranular amorphous or crystalline secondary phases. While such phases may slightly reduce hardness, they can improve fracture toughness through mechanisms such as crack deflection, crack bridging, and limited grain-boundary sliding. Consequently, LPS-processed SiC typically exhibits a balanced combination of high density and improved damage tolerance.

For armor ceramics, ballistic performance is strongly influenced by hardness, elastic modulus, and the distribution of microstructural flaws. Fine-grained microstructures with minimal secondary phases generally exhibit higher hardness and improved resistance to projectile penetration. From this perspective, SPS-processed SiC often demonstrates superior hardness and microstructural uniformity, which are advantageous for maximizing ballistic resistance. Nevertheless, LPS remains attractive for industrial production because it enables efficient densification at lower temperatures and offers improved fracture toughness and reliability.

Overall, both SPS and LPS represent the most advanced sintering strategies for SiC armor ceramics. SPS is generally associated with higher hardness and refined microstructures that favor ballistic resistance, whereas LPS offers improved processing scalability and enhanced damage tolerance due to the presence of grain-boundary phases.

## 4. Enhancing the Armor Properties by Additives

The mechanical properties of SiC are influenced by the microstructure of sintered SiC. Several factors can affect the microstructure, such as the starting materials [41,42,43,52,58,83,120], sintering additives, and sintering atmosphere [121,122].

Sintering of undoped SiC typically requires high temperatures and the use of sintering additives due to SiC’s extremely low self-diffusion coefficient, which makes densification possible only at very high temperatures (above 2100 °C) and/or under high pressure.

To suppress excessive grain growth during sintering, the incorporation of reinforcing phases into a ceramic matrix has proven effective. These reinforcements help by lowering the sintering temperature and enhancing the overall material properties. However, not all reinforcing phases are beneficial—some with inferior properties compared to the matrix can degrade the final composite’s performance. In contrast, certain reinforcements play a crucial role in enhancing densification at relatively lower temperatures by accelerating the shrinkage rate and promoting the formation of dense, fine-grained microstructures, thereby improving the mechanical strength of the sintered body.

As was mentioned above, the residual stress, grain size, porosity, and pore size strongly influence the strength of anisotropic (non-cubic) structural ceramics, especially SiC. Thus, controlling the grain size and the density of SiC in relation to the volume fraction of reinforcement is essential for strengthening the composite. Selecting a suitable reinforcing phase and an appropriate sintering process is critical for material optimization. This section is dedicated to the influence of the reinforcing phase for the consolidation of SiC materials.

Sintering additives might enhance β-to-α-SiC transformation. β-SiC is metastable at 1600–1900 °C and might transform to α-SiC. The β-to-α-SiC transformation depends on several factors, such as temperature, pressure, sintering additive and sintering atmosphere.

### 4.1. Boron, Carbon and Metal Additives

In SSS, as was mentioned earlier, the most widely used additive is elemental boron, which, however, is effective only in conjunction with carbon [20,24,46,65].

Boron and carbon are typically introduced in the amounts of 0.5 and 3.0 wt% [24,52,111,117,123,124,125], respectively, since high-temperature stability is required of such a composition (Table 5).

Furthermore, Al, beryllium and magnesium [72,73,126,127,128] have also been employed as sintering additives. In some cases, these additives facilitate densification via transient liquid-phase sintering or activated sintering pathways, rather than through conventional solid-state diffusion mechanisms.

The role of Al as a sintering aid for SiC was initially identified by He et al. [72] who found that Al was the most effective additive after boron. Building on this, Stutz et al. [73] explored the sintering behavior of β-SiC using combinations of boron with carbon and Al with C.

Tanaka et al. [126] were the first to demonstrate PLS sintering of SiC using aluminum–carbon additives. They observed that the densification mechanism resembled that of the boron–carbon system. Aluminum–carbon additives were thought to facilitate densification at grain boundaries. However, a higher amount of Al was needed compared to the B-C system, primarily because Al has a greater solubility in SiC than boron, and due to the volatilization of reaction products like Al_4_SiC_4_ and Al_4_C_3_ during sintering. Further comparative analysis by Stutz et al. [73] indicated that under certain conditions, aluminum–boron–carbon additive systems could outperform pure boron-based additives in promoting densification. Ghezelbash et al. [127] conducted PLS sintering at 1950 °C for 1 h under an argon atmosphere using different amounts of Al (1–15%) and confirmed by X-ray diffraction the formation of the Al_4_SiC_4_ phase, along with demonstrating that the addition of Al not only improves sinterability at a lower temperature but also enhances the densification and hardness of SiC (3.05 g/cm^3^ and 26.9 GPa respectively).

In LPS, the formation of a liquid phase is critical, and the selection of sintering additives is typically governed by the eutectic characteristics of two or more constituent materials.

In systems employing a single sintering aid, the liquid phase often arises from a eutectic reaction between the native SiO_2_ layer present on SiC surfaces and the additive. Given the highly negative Gibbs free energy of formation for SiO_2_ (~785 kJ/mol), the spontaneous formation of a SiO_2_ film on SiC is thermodynamically favored under ambient conditions [128].

Noviyanto and Yoon [16] reported the existence of an ultra-thin SiO_2_ film (<2 nm) on fine-grained β-SiC. The enhancement of SiC’s relative density is closely linked to the wetting behavior of the liquid phase, with lower contact angles promoting improved densification. Liu et al. [129] investigated the wettability of various metals on SiC and found that the native SiO_2_ layer impedes both adhesion and wetting of molten metals. Consequently, the removal or reduction of this oxide layer is proposed to enhance wettability. Binary alloys such as Au–Si have exhibited favorable wetting angles, contributing to improved interfacial interactions. Proper wettability is crucial not only for densification but also for preserving the mechanical integrity of ceramics during service. In [129], they confirmed that Au, Ag, Sn, Pb, and Ge remain non-reactive with SiC at elevated temperatures.

Negita [14] produced a guideline for effective sintering additives for SiC that are deemed thermodynamically favorable based on the Gibbs free energy. At 2127 °C, metals such as Al, B, Co, Fe, Li, Mg, and Ni qualify, though Li and Mg require stabilization due to their volatility at high temperatures.

Lin et al. [130] demonstrated successful HP of β-SiC with Al-B-C additives at 1600–1800 °C under 20–60 MPa.

Noviyanto and Yoon [16] assessed the reactivity of β-SiC with metals like Mg, Al, Ti, V, Cr, Fe, Ta, and W. Their thermodynamic and experimental analyses concluded that Al and Mg are effective sintering additives that significantly enhance densification without decomposing β-SiC. Al, in particular, achieved 96.1% and 99.0% density at 5 and 10 wt.%, respectively. In contrast, Cr, Fe, Ta, Ti, V, and W, despite being safe elements under neutron irradiation conditions [131], formed metal carbides or silicides by breaking down SiC. Though β-SiC was used, a transformation to α-SiC was observed at high temperatures. Traces of Al_4.95_Si_1.05_O_9.52_ and Mg_2_SiO_4_ were found due to reactions between the additives and SiO_2_ adsorbed on SiC. For Cr, the formation of Cr_7_C_3_, Cr_5_Si_3_, and free carbon was noted. Fe produced FeSi, while Ta, Ti, and V generated only their corresponding carbides alongside α- and β-SiC. W formed both W_2_C and W_5_Si_3_, aligning with predictions based on standard Gibbs energy and vapor pressures. Grain size analysis revealed that samples with ineffective additives (Cr, Fe, Ta, Ti, V, W) had significant growth, reaching 2 μm higher than the original β-SiC particle size of 52 nm. In contrast, Al- and Mg-containing samples retained fine grain sizes under 200 nm. Coarse grains are typical of SSS SiC, while liquid-phase sintered SiC tends to exhibit finer, more uniform grains. This suggests that Al and Mg promote liquid-phase formation through reactions with SiC and surface SiO_2_, facilitating efficient mass transport and densification.

In a separate study, Li et al. [96] investigated SiC ceramics fabricated via HP with the addition of approximately 7.8 wt% magnesium and 0.2 wt% Al. The resulting materials exhibited improved toughness (4.6 MPa·m^1/2^), though lower flexural strength (172.3 MPa) and hardness (up to 6.7 GPa) compared to most of the other reinforced variants.

### 4.2. Oxides

The concept of using metal oxides as sintering additives for SiC densification through LPS dates back to the work of Liu et al. [90], who demonstrated its effectiveness under PLS sintering conditions. Building on this, Negita [14] conducted thermodynamic evaluations to identify suitable oxide additives—particularly metal and rare-earth oxides—that remain chemically stable with SiC at sintering temperatures, making them promising candidates for LPS-based SiC fabrication.

One of the primary challenges in incorporating metal oxides into ceramic matrices is their tendency to engage in complex chemical reactions at elevated sintering temperatures. Studies by Negita [14] reported that metal oxides can react with SiC through various oxidation pathways, often leading to the formation of gaseous by-products. These reactions result in significant weight loss and structural changes during thermal treatment. By analyzing the standard Gibbs free energy and vapor pressures at temperatures exceeding 2000 °C, Negita [14] identified Al_2_O_3_, BeO, HfO_2_, and Y_2_O_3_ as effective sintering additives that do not induce the decomposition of SiC under high-temperature conditions.

Noviyanto and Yoon [17] evaluated several metal oxides—namely Al_2_O_3_, Fe_2_O_3_, MgO, TiO_2_, WO_3_, and Y_2_O_3_—for their reactivity and densification behavior when used as sintering aids for β-SiC. Their study combined thermodynamic modeling with HP experiments conducted at 1700–1800 °C. The results revealed that Al_2_O_3_, MgO, and Y_2_O_3_—either individually or in mixed systems—acted as suitable sintering additives, maintaining the stability of β-SiC. Conversely, Fe_2_O_3_, TiO_2_, WO_3_, and their combinations exhibited undesirable reactivity with SiC, forming metal carbides or silicides, consistent with thermodynamic predictions. It is worth noting that TiO_2_ in combination with Al_2_O_3_ and Y_2_O_3_ [30,115,132,133] effectively suppressed excessive grain growth in the SiC matrix, and improves mechanical properties of the product.

A distinct microstructural evolution and sintering mechanism was observed with the addition of Y_2_O_3_, as reported by Noviyanto and Yoon [17]. Unlike other additives that promote the decomposition of SiC into metals, metal carbides, or silicides, Y_2_O_3_ maintains the integrity of SiC. Instead, it expected to react with the SiO_2_ layer present on the SiC surface, forming a transient liquid phase. This reaction initiates the first stage of LPS, characterized by particle rearrangement, which was confirmed by achieving relative densities exceeding 90%. However, the efficiency of this rearrangement is influenced by factors such as the solid solubility of SiC in the liquid phase and the wetting angle between the liquid and SiC surfaces.

Taguchi et al. [134] investigated the wetting behavior of binary oxide systems such as Y_2_O_3_–Al_2_O_3_ and Sm_2_O_3_–Al_2_O_3_ on SiC surfaces. Their study revealed that contact angles decreased with increasing temperature, reaching their lowest values at eutectic compositions, and processing in argon resulted in better wettability compared to nitrogen, because bubbles formed in the liquid during the test with N_2_.

To date, several common additives, such as Al_2_O_3_–Y_2_O_3_ [29,32,42,58,79,80,83,84,85,86,89,97], Al_2_O_3_ alone or with other oxides [25,135,136], and Al_2_O_3_–Y_2_O_3_–CaO [11,81,82], have demonstrated high efficiency in enhancing the densification, microstructural features and mechanical properties, including hardness, flexural strength, and fracture toughness, of SiC ceramics.

While Al_2_O_3_–Y_2_O_3_ combinations are widely recognized as effective sintering additives, the incorporation of Al_2_O_3_ with various rare-earth oxides has enabled tailored microstructural development, thereby refining the mechanical and physical properties of SiC ceramics [87,88].

The influence of the rare-earth cation size on the secondary phase characteristics and the sintering behavior of liquid-phase sintered SiC has also been systematically studied [100]. Sintering was performed at 2000 °C for 1 h under a slight N_2_ overpressure of 0.2 MPa for 30 min, followed by a pressure sintering cycle of 30 min at the same temperature under 10 MPa N_2_ to achieve complete densification. Samples with Gd_2_O_3_, Ho_2_O_3_, Dy_2_O_3_, Y_2_O_3_, and Lu_2_O_3_ along with AlN additives were prepared, and ceramics with high mechanical properties (3.5–5.0 MPa·m^1/2^ fracture toughness, 20.0–22.5 GPa hardness) were obtained. These investigations revealed a strong dependence of the microstructure and densification behavior on the ionic radius of the rare-earth additives; the element with a higher cationic radius (Dy^3+^) segregates at intergranular films, whereas the content of the element with a lower atomic radius (Ho^3+^) is found to be higher at triple points.

In an extensive thermodynamic and experimental evaluation, Noviyanto and Yoon [18] assessed a wide range of rare-earth oxides, including Sc_2_O_3_, CeO_2_, Nd_2_O_3_, Sm_2_O_3_, Gd_2_O_3_, Dy_2_O_3_, Ho_2_O_3_, Er_2_O_3_, La_2_O_3_, Tm_2_O_3_, Yb_2_O_3_, and Lu_2_O_3_, as potential sintering aids for β-SiC. Their work highlighted that PLS liquid-phase sintering using combinations of Al_2_O_3_ with selected rare-earth oxides (e.g., Lu_2_O_3_, Er_2_O_3_) leads to enhanced densification and structural stability of SiC ceramics. They also reported that rare-earth oxides can form eutectic systems with surface SiO_2_ at temperatures below 1700 °C, contributing to the formation of a transient liquid phase essential for sintering. Moreover, the presence of rare-earth oxides was found to promote the phase transformation from β-SiC to α-SiC during the sintering process.

Liang and co-workers [29] explored the effects of various rare-earth oxide additions (Lu_2_O_3_, Er_2_O_3_, and Ce_2_O_3_) on the mechanical properties, thermal conductivity, and microstructure of α-SiC. The mechanical properties of these ceramics was related to the ionic radius of the rare-earth cations, i.e., a decrease in the cationic radius of RE_2_O_3_ was accompanied by an increase in hardness (23.0–27.6 GPa) and flexural strength (437–565 MPa) and a decrease in fracture toughness (4.6–4.1 MPa·m^1/2^) of SiC ceramics, and vice versa.

As previously mentioned, the densification and mechanical properties of SiC ceramics are significantly influenced by the particle size of both the primary powder and the sintering additives. Noviyanto and Yoon [18] investigated the effect of additive particle size on the sintered density of SiC. By comparing fracture surfaces of SiC samples containing 5 wt.% of rare-earth oxides in both fine and coarse forms, they found that the finer additive led to higher densification and coarser SiC grains. This enhancement was attributed to improved homogeneity and distribution of the additive within the matrix, where a 3–8% increase in relative density was observed

The literature [11,25,29,30,32,42,58,79,80,81,82,83,84,85,86,87,88,97,115,120,122,133,134,135,136,137] highlights various sintering methods and additive systems with metal oxides that significantly enhance the mechanical properties of SiC ceramics (Table 6). According to compiled data, Al_2_O_3_–Y_2_O_3_ combinations remain the most widely used sintering additives, sometimes supplemented with minor oxide additives.

For instance, Lee [82] achieved a remarkable fracture toughness of 8.4 MPa·m^1/2^ using a two-step sintering process (HP at 25 MPa and 1800 °C, followed by PLS sintering at 1950 °C in Ar) with a ternary additive system composed of 5.7 wt% Al_2_O_3_, 3.3 wt% Y_2_O_3_, and 1 wt% CaO.

Similarly, Kornaus et al. [95] reported the production of SiC ceramics with a high flexural strength of 715 MPa and a fracture toughness of 5.3 MPa·m^1/2^ using HP at 2000 °C and 25 MPa with 8.2 wt% of an Al_2_O_3_/Y_2_O_3_ mixture (mass ratio 3:2).

High-hardness ceramics, reaching 30 GPa, have been reported by Abilev et al. [118] through liquid-phase UHPS at 1800 °C under 5000 MPa, using 2.5 wt% MnO, 2.0 wt% Al_2_O_3_, and 4.5 wt% SiO_2_. Further, Zhambakin et al. [119] achieved 31–38 GPa hardness via SPS at 2000 °C and 19 MPa with a similar additive composition, changing part of the starting SiC powder to a finer grade (280 nm in average).

Zhou et al. [88] explored the role of rare-earth oxides (La_2_O_3_, Nd_2_O_3_, Y_2_O_3_, Yb_2_O_3_) in combination with Al_2_O_3_, sintering SiC at 1800 °C and 40 MPa followed by annealing at 1950 °C for 3 h. The resulting ceramics exhibited fracture toughness values of 3.4–5.5 MPa·m^1/2^ and flexural strengths of 587–661 MPa.

Ciudad et al. [87] achieved fully dense SiC ceramics via PLS sintering at 1950 °C, incorporating 10 vol.% of Al_2_O_3_ and RE_2_O_3_ (La, Yb, or Nd) in a 5:3 ratio. The materials exhibited fracture toughness in the range of 4.0–4.4 MPa·m^1/2^, flexural strength of 365–405 MPa, and hardness between 17.2 and 21.4 GPa.

Khodaei et al. [30] investigated the effect of incorporating nano-sized TiO_2_ particles, alongside conventional Al_2_O_3_ and Y_2_O_3_ sintering additives, on the mechanical performance of SiC matrix composites. The composites were fabricated via PLS sintering at 1900 °C. Their findings revealed that adding up to 4.5 wt% TiO_2_ effectively suppressed excessive grain growth in the SiC matrix. The microstructure, as well as the final mechanical properties, was significantly influenced by the densification level, the phases formed during sintering, and their distribution within the matrix. The optimal properties—including a high relative density of 98.7%, Young’s modulus of 401.2 GPa, hardness of 27.1 GPa, indentation fracture resistance of 6.1 MPa·m^1/2^, flexural strength of 522.7 MPa, and the lowest brittleness index of 292.19 × 10^−6^ m^−1^—were achieved in the sample with 4.5 wt% TiO_2_. These improvements were attributed to residual stresses from the mismatch in thermal expansion coefficients between in situ-formed TiC and the SiC matrix, in addition to toughening mechanisms such as microcracking, crack deflection, crack branching, and grain pull-out.

In a related study, Khodaei et al. [135] examined the impact of Cr_2_O_3_ nanoparticle additions on the sintering behavior and mechanical properties of SiC–Al_2_O_3_–Y_2_O_3_ ceramics. Microstructural analysis revealed the presence of yttrium aluminum garnet, yttrium aluminum monoclinic, Cr_2_O_3_, and residual SiC phases. The sample with 5 wt% Cr_2_O_3_ exhibited the best performance, achieving 97.45% relative density, 28.19 GPa hardness, 403 GPa elastic modulus, and 6.2 MPa·m^1/2^ fracture toughness. It was observed that the amount of Cr_2_O_3_ played a critical role in controlling the SiC grain size and densification. At higher Cr_2_O_3_ concentrations, grain growth was promoted, which, while beneficial to toughness through mechanisms such as crack bridging, deflection, and branching, could also lead to a reduction in fracture toughness due to increased grain aspect ratios beyond the optimum.

Shinoda et al. [115] employed SPS to produce nanocrystalline SiC ceramics with Al_2_O_3_ and TiO_2_ additives. Their study confirmed the in situ formation of TiC due to the reaction between TiO_2_ and SiC during sintering, with Al_2_O_3_ used to enhance the densification and suppress exaggerated grain growth.

Liang et al. [133] further explored the in situ formation of TiC within a SiC matrix by utilizing TiO_2_ as a reactive Ti source. The resulting composites demonstrated higher densification, flexural strength, Vickers hardness, and fracture toughness compared to those made with pre-formed TiC. These enhancements were credited to the refined microstructure and improved particle–matrix interface bonding enabled by the in situ synthesis process.

### 4.3. Various Reinforcing Phases

In this section, we observe the role of various reinforcing sintering additives for SiC ceramics—in some cases, along with those discussed in previous sections—focusing on those which enhance mechanical properties. Besides the commonly used metal oxides—often applied in combination—additives such as titanium carbide (TiC), titanium diboride (TiB_2_), boron carbide (B_4_C), chromium carbide (Cr_3_C_2_), aluminum nitride (AlN), oxynitrides, and metal nitrates have demonstrated effectiveness in enhancing densification and mechanical performance (Table 7).

Nitrates, in particular, have emerged as promising sintering additives due to their ease of incorporation into liquid-phase sintering systems and their in situ transformation into oxides during calcination. A notable method was reported by Ortiz et al. and Borrero-Lopez et al. [121,136], who developed a colloidal processing route for PLS sintering of SiC using a low content of sintering additives. This method involved depositing a sol–gel-derived additive precursor onto SiC particle surfaces, achieving uniform additive dispersion within the green body, thus promoting effective densification.

Huang et al. [137] investigated the mechanical behavior of SiC ceramics sintered with 9 wt.% of a nitrate mixture (Mg(NO_3_)_2_, Al(NO_3_)_3_, and Y(NO_3_)_3_) and compared the results with those obtained using oxide additives. They reported a significant increase in fracture toughness from 3.47 to 5.76 MPa·m^1/2^ when using nitrates, although this improvement came with a reduction in hardness from 22.5 to 11.0 GPa.

Further research by Noviyanto et al. [138] systematically evaluated fourteen rare-earth metal nitrates as sintering aids for β-SiC. Nitrate quantities were calculated to correspond to 5 wt.% of the oxide form. In particular, nitrates of Sc, Yb, Tm, Er, and Ho yielded sintered bodies with >99% relative density. No residual rare-earth metals, carbides, or silicides were detected, indicating the thermal compatibility of rare-earth nitrates with SiC and their potential for inducing beneficial liquid-phase formation during sintering. However, other parameters such as the volume fraction and wettability are also critical to their performance.

AlN has emerged as an effective sintering additive for SiC ceramics, facilitating densification through the formation of transient liquid phases during high-temperature sintering. Its addition promotes improved mechanical properties and contributes to microstructural refinement as well as grain growth inhibition. Although AlN alone may not produce sufficient liquid phase for optimal densification, it demonstrates excellent synergistic effects when combined with other additives such as Y_2_O_3_ or Al_2_O_3_, enabling near-theoretical densities via PLS sintering.

In 1991, Wei and Lee [139] successfully achieved PLS sintering of SiC–AlN composites by incorporating AlN alongside yttria (Y_2_O_3_) as sintering aids. This combination facilitated the formation of a Y–Al–Si–O–N grain-boundary phase, which significantly enhanced densification at sintering temperatures between 2050 and 2100 °C.

Subsequent studies have built upon this foundational work. For instance, research by Ordan’yan and Vikhman [140] explored the SiC–AlN system, demonstrating that ceramics containing 20–30 vol% AlN and heat-treated at 2100 °C exhibited structural transformations that increased the material’s strength to 800–900 MPa.

Strecker et al. [141] studied how the sintering time and raw material ratios affect the fracture toughness of PLS liquid-phase sintered SiC composites using Y_2_O_3_ and AlN as secondary phases. Samples were sintered at 2080 °C for 1 h, with some undergoing post-sintering annealing at 2000 °C for 4 h. Results showed that a higher AlN content led to better densification. However, annealing reduced densification in samples with low AlN but had no effect on those with high AlN. This behavior is attributed to AlN-rich phases being prone to weight loss via evaporation or reaction, though AlN rarely interacts with SiC in N_2_ atmospheres [142]. Increasing AlN also promoted elongated SiC grains with high aspect ratios, enhancing fracture toughness (6.7 MPa·m^1/2^) and hardness (9.5–18.2 GPa).

Previously Biswas et al. [142] have also investigated the role of AlN as a sintering additive and the phase composition in tailoring the mechanical properties of SiC-based ceramics by PLS sintering using 10 vol% AlN/Y_2_O_3_ in combination with varying proportions of starting powders and annealing times. The resulting ceramics possess high mechanical properties, such as fracture toughness values ranging from 4.9 to 6.1 MPa·m^1/2^ and flexural strength between 480 and 520 MPa.

Izhevskyi et al. [43] achieved similar results using GP sintering at 1950 °C with 10.65 wt% Y_2_O_3_ and 2.9 wt% AlN. Their work focused on the impact of the SiC particle size, with the researchers reporting fracture toughness values between 3.7 and 5.9 MPa·m^1/2^ and hardness in the range of 17.5 to 21.0 GPa depending on the grain size of α-SiC—the finer, the better.

Eom et al. [26] conducted PLS sintering at 1900 °C with various combinations of Al_2_O_3_, Y_2_O_3_, and AlN as additives. They achieved high fracture toughness (6.5–6.9 MPa·m^1/2^) and flexural strength (399–425 MPa). Interestingly, even in the absence of AlN, similar toughness (~6.8 MPa·m^1/2^) and a slightly higher strength (415 MPa) were observed, suggesting AlN’s limited role in toughness enhancement under their conditions. The authors suggest that the glass compositions of the SiC-Al_2_O_3_-Y_2_O_3_ and SiC-Al_2_O_3_-Y_2_O_3_-AlN specimens were an oxide glass and an oxynitride glass, respectively. The further addition of nitrogen (higher wt% of AlN) decreased the fracture toughness slightly from 6.9 to 6.5 MPa·m^1/2^, suggesting that the addition of nitrogen beyond the solubility limit of nitrogen in an oxynitride glass is not beneficial in improving the toughness of LPS SiC ceramics [26].

Kim et al. [143] performed a comparable experiment using a starting α-SiC phase instead of β-SiC under PLS sintering at 1900 °C with 0.86 wt% Al_2_O_3_, 1.9 wt% Y_2_O_3_, and 1.04 wt% AlN. Their results indicated a lower fracture toughness of 3.1 MPa·m^1/2^ but higher flexural strength of 582 MPa and increased hardness up to 25.7 GPa, highlighting the influence of the initial SiC polymorph on mechanical properties.

Cho et al. [144] compared α-SiC and β-SiC systems using Y_2_O_3_, Sc_2_O_3_, and AlN additives. Mechanical properties, such as fracture toughness and flexural strength, improved from 4.1 MPa·m^1/2^ and 509 MPa with α-SiC to 5.1 MPa·m^1/2^ and 520 MPa with β-SiC, although hardness decreased slightly from 27.2 to 25.0 GPa. These results reinforce the importance of the SiC polytype and additive selection in optimizing composite performance.

Along with AlN, from nitrides, titanium nitride (TiN) is a promising sintering additive for SiC ceramics in armor applications due to its ability to enhance densification and mechanical performance. TiN promotes liquid-phase formation during sintering, improving particle rearrangement and densification at lower temperatures. It also acts as a grain growth inhibitor, refining the microstructure and increasing fracture toughness through mechanisms such as crack deflection and microcracking. However, TiN has limited sinterability on its own and requires oxide additives or advanced techniques. It can also react under oxidizing or nitrogen-deficient conditions, leading to undesirable phases. Additionally, thermal expansion mismatch between TiN and SiC may cause residual stresses and microcracking.

As a notable example, Guo et al. [145] investigated the effect of nano-TiN additions on the sintering behavior and mechanical properties of SiC ceramics. They found that incorporating 5 wt.% nano-TiN led to high densification, a uniform microstructure, and enhanced mechanical properties (7.04 MPa·m^1/2^ fracture toughness, 686.8 MPa strength). The toughening mechanisms were attributed to thermal residual stresses, crack deflection, and crack bridging.

Oxynitride-based sintering additives have shown notable potential for enhancing the mechanical properties of SiC ceramics. Lee et al. [82] reported that the addition of 10 wt.% Y-Mg-Si-Al-O-N oxynitride to β-SiC, by HP at 1800 °C under 25 MPa for 1 h, resulted in near-full densification (≥99.9%). In comparison, using 10 wt.% Al_2_O_3_-Y_2_O_3_–CaO yielded a lower relative density of 97%. However, despite the superior densification, the fracture toughness was higher in oxide-doped samples—ranging from 5.9 to 8.4 MPa·m^1/2^—while oxynitride-doped samples exhibited values between 3.4 and 3.8 MPa·m^1/2^, depending on the dopant content. No hardness data was provided, though it is likely that oxynitride additions could lead to increased hardness.

An and Kim [89] examined SiC ceramics sintered by HP at 1820 °C for 1 h under 25MPa, followed by post-annealing at 1930 °C for 6 h. They used TiC as the primary secondary phase and varied the sintering additives: Al_2_O_3_ + Y_2_O_3_, Y_2_O_3_ + AlN, and oxynitride. High relative densities of 96.6–97.6% were achieved across all systems. Fracture toughness varied with the additive type, from 5.9 MPa·m^1/2^ (Y_2_O_3_ + AlN) to 6.5 MPa·m^1/2^ (oxynitride), and peaking at 7.4 MPa·m^1/2^ with 3 wt.% Al_2_O_3_ + 7 wt.% Y_2_O_3_.

As it is described in a previous example [89], incorporating titanium carbide (TiC) into SiC matrices has been shown to improve fracture toughness, flexural strength, and hardness through mechanisms such as crack deflection, grain refinement, and residual stress toughening. TiC has been extensively studied as a sintering additive for SiC ceramics, as well as aiming to enhance mechanical properties critical for armor applications. These enhancements are primarily due to the uniform dispersion of TiC particles, which impede crack propagation and refine the microstructure, leading to an improved mechanical performance. The synergistic effects of TiC addition and advanced sintering techniques underscore the potential of SiC-TiC composites in high-performance armor systems.

Luo et al. [116] fabricated SiC ceramics by SPS at 1800 °C for 20 min, applying 40 MPa with only TiC in amounts of 15 and 30%. The samples with 30% TiC possess 6.25 MPa·m^1/2^ fracture toughness, 670 MPa strength and 28 GPa hardness, while samples with 15% TiC have half the fracture toughness and flexural strength. The SEM observation showed that the fracture patterns of composites had two models: intergranular and transgranular fracture.

Shaoming et al. [104] studied the mechanical properties of SiC–TiC composites fabricated via HIP at 1850 °C and 200 MPa, using two types of TiC powders (100 nm and 1000 nm). While the TiC particle size had a negligible effect on flexural strength, a coarse TiC content improved fracture toughness (from 3.97 to 5.13 MPa·m^1/2^), whereas a lower TiC content resulted in slightly greater hardness (22.5 vs. 21.7 GPa).

Chae et al. [93] examined the effect of Cr_3_C_2_ on the sintering behavior of SiC composites reinforced with 30 vol% TiC. The addition of Cr_3_C_2_ enhanced densification and mechanical properties via liquid-phase formation. At 1950 °C with 1 vol% Cr_3_C_2_, 98.5% densification, 750 MPa flexural strength, and 4.6 MPa·m^1/2^ fracture toughness were achieved. Increasing Cr_3_C_2_ to 10 vol% raised the fracture toughness to 6.5 MPa·m^1/2^, attributed to α-SiC grain elongation and β-phase formation.

In a study conducted by Cho et al. [117], SiC-TiC composites were fabricated via SPS using TiC in combination with Al, B, and C as sintering aids. Compositions containing up to 40 wt% TiC achieved full densification at a sintering temperature of 1800 °C. The resulting microstructure exhibited a significant enhancement in mechanical properties, with the optimized composite reaching a fracture toughness of 6.3 MPa·m^1/2^ and a flexural strength of 720 MPa.

Kornaus et al. [95] fabricated hot-pressed SiC ceramics at 2000 °C under a pressure of 25 MPa using submicron SiC powder (800 nm) with varying amounts of TiC and Al_2_O_3_-Y_2_O_3_ (mass ratio 3:2) as sintering additives. A baseline sample containing 8.2 wt% Al_2_O_3_-Y_2_O_3_ exhibited a flexural strength of ~715 MPa, fracture toughness of 5.3 MPa·m^1/2^, and hardness of 18.7 GPa. Substitution with 4.5 wt% TiC and 11 wt% Al_2_O_3_-Y_2_O_3_ slightly reduced strength (~705 MPa) but improved toughness to 6.3 MPa·m^1/2^. Further increasing the TiC content to 12.9 wt% (with 6.5 wt% Al_2_O_3_-Y_2_O_3_) and 24 wt% (with 5.8 wt% Al_2_O_3_-Y_2_O_3_) led to a progressive decline in strength (550 and 475 MPa, respectively), while fracture toughness remained relatively stable (5.9–5.8 MPa·m^1/2^). Notably, hardness increased, with a higher TiC content, reaching 19.7 GPa in the sample with 24 wt% TiC. These results suggest a trade-off between strength and toughness/hardness with an increasing TiC content under identical hot-pressing conditions.

Recently, Ivzhenko et al. [146] studied SiC–TiC, SiC–VC composite ceramics obtained by solid-state electrospark sintering at a pressure of 45 MPa and the temperatures of 1900 and 2000 °C. The addition of 40 vol.% Ti and V carbides to SiC significantly increases densification to 99.9% in the 60SiC–40TiC composite and 91.2% in the 60SiC–40VC composite. Composites with titanium carbide admixture are better than composites with vanadium carbide admixture due to increased interaction at phase boundaries. The interaction zone increases from ~1.0 μm at the boundaries of SiC–VC grains to ~1.5 μm at the boundaries of SiC–TiC grains. Consequently, SiC–VC ceramics possess a fracture toughness of 5.2 MPa·m^1/2^, and hardness of 13.7 GPa, lower than SiC–TiC composites (5.7 MPa·m^1/2^ and 21.5 GPa).

Mostly studies in the literature focus on B_4_C-based ceramics where B_4_C serves as the primary phase, but such systems are beyond the scope of the current discussion. There are some investigations where B_4_C is employed as a sintering aid for SiC ceramics; for example, Feng et al. [41] investigated the effect of adding 1.5 wt% B_4_C and 3.5 wt% carbon to purified SiC powders, which were densified via PLS sintering at 2000 °C. The resulting ceramics exhibited enhanced mechanical properties, achieving a fracture toughness of 5.1 MPa·m^1/2^, a hardness of 27 GPa, and a flexural strength in the range of 300–315 MPa.

In another study, Bahaaddini et al. [147] evaluated the influence of B_4_C additions (1–6 wt%) on PLS-sintered SiC ceramics with a fixed rest additives of 1 wt% C, 5.98 wt% Y_2_O_3_, and 4.02 wt% Al_2_O_3_ at 1900 °C. They observed that increasing the B_4_C content up to 5 wt% led to a significant improvement in fracture toughness from 2.78 MPa·m^1/2^ (0 wt% B_4_C) to 5.87 MPa·m^1/2^, while hardness concurrently increased from 9.58 GPa to 22.88 GPa—demonstrating no apparent trade-off between these two properties. The improved mechanical performance was attributed to B_4_C’s role in suppressing SiC grain growth and initiating porosity to decrease, consequently increasing density. Authors suggested that crack growth deflection by collision with or impediment to the crack path are the main mechanisms that increase the hardness and toughness of the samples.

Titanium diboride (TiB_2_) is a valuable additive in the sintering of SiC ceramics, enhancing densification and mechanical properties. TiB_2_ particles inhibit grain growth in SiC by pinning grain boundaries, leading to a finer microstructure. TiB_2_ promotes densification during sintering, especially when combined with additives like AlN and Y_2_O_3_. These additives can react to form intergranular phases such as Y_4_Al_4_O_9_, which facilitate liquid-phase sintering and improve the packing of SiC grains. As for challenges, using TiB_2_ is a sintering difficulty due to the high melting point (~3225 °C) and strong covalent bonding, making it challenging to sinter without additives or advanced techniques like SPS. Moreover, there is a risk of oxide layer formation (e.g., TiO_2_ and B_2_O_3_) on TiB_2_ particles, which could hinder sintering by promoting grain growth and porosity.

Grigoriev et al. [44] fabricated SiC-based ceramic composites reinforced with TiB_2_ and TiC via SPS at 2000 °C under a vacuum and an applied pressure of 80 MPa for 3 min. and TiC. The composition with 80 vol% SiC–12.5 vol% TiB_2_–7.5 vol% TiC exhibited a fracture toughness of 6 MPa·m^1/2^, flexural strength of 511 MPa, and Vickers hardness of 20.4 GPa, while the composition with 60 vol% SiC–25 vol% TiB_2_–15 vol% TiC showed enhanced mechanical properties, with a fracture toughness of 6.2 MPa·m^1/2^, strength of 588 MPa, and hardness of 22.9 GPa. The improved toughness in the 60SiC composite was attributed to mechanisms such as crack deflection at TiB_2_ and TiC grains, crack branching, and interfacial debonding. Both composites exhibited a combination of transgranular and intergranular fracture modes within the SiC matrix.

In the study by Cho et al. [148], SiC-based composites were reinforced with TiC and TiB_2_ and consolidated through HP at 1850 °C for 1 h under a pressure of 25 MPa. The incorporation of TiB_2_ and TiC as secondary phases significantly enhanced the fracture toughness of the composites, although a slight reduction in flexural strength was observed. Specifically, the addition of 30–50 wt% TiB_2_ and 30–50 wt% TiC increased the fracture toughness of SiC from approximately 3 MPa·m1/2 to 4.1–4.5 MPa·m^1/2^. Microstructural examination of the SiC–TiB_2_ composites indicated that the primary toughening mechanisms were microcrack formation and crack deflection around the reinforcing particles. Despite the modest decline in strength, the improved toughness underscores the beneficial role of TiB_2_ and TiC in enhancing the mechanical performance of SiC ceramics.

Li et al. [98] synthesized SiC ceramic using internally synthesized TiB_2_ from TiN, as a reinforcing phase. The composites, containing 10, 20, and 30 vol% TiB_2_, were consolidated via HP at 2080 °C under a pressure of 20 MPa. Among these, the composite with 20 vol% TiB_2_ demonstrated a notable enhancement in fracture toughness—approximately an 80% increase compared to monolithic SiC—while maintaining a comparable flexural strength of around 490 MPa. This indicates that the in situ formation of TiB_2_ can effectively improve the toughness of SiC without compromising its strength.

In the study by Blanc et al. [149], SiC–TiB_2_ composites were fabricated via PLS sintering at 2190 °C using submicron SiC powders (700 nm) and varying TiB_2_ contents (5–15 vol%). The TiB_2_ particles were homogeneously distributed within the SiC matrix. A slight but consistent increase in fracture toughness was observed with an increasing TiB_2_ content, attributed to crack deflection around the dispersed particles. Specifically, fracture toughness values increased from 3.5 MPa·m^1/2^ (5 vol%) to 3.9 MPa·m^1/2^ (15 vol%), while hardness decreased from 30 GPa to 23 GPa over the same range.

In a similar study by Wang et al. [150], a finer SiC grade (400 nm) and in situ synthesized TiB_2_ were used, with additives such as B_4_C, C, and cost-effective TiO_2_. The composites were PLS sintered at 2000 °C, with a pre-sintering step at 1400 °C to ensure complete conversion of TiO_2_ to TiB_2_. The optimal carbon content was determined to be 4.0 wt% beyond reaction requirements. Compared to the Blanc et al.’s [149] study, significantly higher fracture toughness values were achieved—rising from 4.9 MPa·m^1/2^ (5 vol%) to 5.9 MPa·m^1/2^ (20 vol%), and further to 6.3 MPa·m^1/2^ at 2100 °C for 15 vol% TiB_2_. The use of micron-sized TiO_2_ powders was found to enhance densification. These results demonstrate that in situ synthesized TiB_2_, combined with optimized processing parameters and finer SiC, can lead to substantial improvements in the fracture toughness of SiC-based composites.

Tani et al. [105] developed SiC matrix composites through the in situ formation of TiB_2_ and carbide phases via carbothermal reduction of oxide precursors. The composites incorporated with B_4_C, TiO_2_, and carbon from various sources to synthesize TiB_2_ within the matrix. These materials were densified using PLS sintering followed by HIP. The HIPed composites sintered at 2000–2100 °C exhibited improved fracture toughness values of 4.7–4.8 MPa·m^1/2^ compared to 2.7 MPa·m^1/2^ for monolithic SiC. Further sintering at 2200 °C increased toughness to 6.2 MPa·m^1/2^, attributed to extensive crack deflection induced by coarsened TiB_2_ grains. Notably, using phenol resin as a carbon source enhanced the mechanical performance, achieving a four-point flexural strength exceeding 800 MPa. The TiB_2_ grain size was found to depend on the sintering temperature, and HIP at 1900 °C primarily eliminated porosity without altering the microstructure.

Cho et al. [92] demonstrated in situ toughening of SiC–30 wt% TiB_2_ composites using liquid-phase sintering with 7 wt% Al_2_O_3_ and 3 wt% Y_2_O_3_ additives. The composites were hot-pressed at 1850 °C and subsequently annealed at 1950 °C. The annealing process promoted the transformation of β-SiC to elongated α-SiC grains and coarsening of TiB_2_, both of which contributed to toughening mechanisms such as crack bridging and deflection. Without annealing, the composite showed a fracture toughness of 4.4 MPa·m^1/2^ and flexural strength of 571 MPa. After 6 h of annealing, the toughness increased to 6.7 MPa·m^1/2^ with a slight reduction in strength to 550 MPa, while 12 h of annealing resulted in 6.4 MPa·m^1/2^ toughness and further strength reduction to 501 MPa. These results highlight the effectiveness of controlled grain growth and phase transformation in improving the toughness of SiC-based composites.

In the study by Gong [27], SiC-based multiphase ceramics were synthesized using SPS with AlN and TiB_2_ as reinforcing phases and Y_2_O_3_ as a sintering aid. The influence of the TiB_2_ content (10 and 20 vol%) and sintering temperatures ranging from 1900 °C to 2100 °C was examined in relation to the materials’ phase composition, microstructure, and mechanical and tribological properties. During sintering, Y_2_O_3_ reacted with Al_2_O_3_ present on the AlN particle surfaces to form Y_4_Al_2_O_9_, a secondary intergranular phase that enhanced densification. The composite containing 10 vol% TiB_2_ exhibited the most favorable mechanical properties, achieving a density of 98.3%, hardness of 28 GPa, fracture toughness of 5.7 MPa·m^1/2^, and bending strength of 553 MPa.

Bucevac et al. [151,152] investigated the in situ formation of TiB_2_ as a reinforcing phase in SiC composites. Fine β-SiC powders (average size 700 nm) were used as the matrix, with Y_2_O_3_ and Al_2_O_3_ serving as liquid-phase sintering additives and B_4_C, TiO_2_, and C as reactants to generate TiB_2_ in situ. The composites were fabricated via PLS sintering at the temperature of 1940 °C for 1 h. Fracture toughness was closely linked to the TiB_2_ content; 30 vol% TiB_2_ yielded the highest toughness of 5.7 MPa·m^1/2^. The presence of TiB_2_ also contributed to grain growth inhibition in the SiC matrix. Densification improved with the TiB_2_ content up to 18 vol%, after which it declined due to porosity formation associated with excess in situ TiB_2_ development. These findings underscore the dual role of TiB_2_ in enhancing toughness and influencing microstructural evolution in SiC composites.

A noteworthy contribution was made by Li et al. in their study on the enhancement of SiC ceramics through the incorporation of Ni_3_Al and Mg_2_Si additives [153]. When utilizing SPS at 50 MPa with the relatively low temperature of 1300 °C, the authors successfully achieved densification of the SiC ceramics. The addition of 10 wt% Ni_3_Al led to substantial improvements in mechanical properties, increasing the microhardness from 4.95 GPa to 13.59 GPa and the fracture toughness from 8.17 to 10.40 MPa·m^1/2^. These enhancements correspond to improvements of 174.55% and 27.29%, respectively. Despite these advances, the resulting hardness remains insufficient for demanding structural applications such as armor, underscoring the need for further optimization.

In the development of armor-grade SiC ceramics, sintering additives should be considered not merely as auxiliary components enabling densification, but as active microstructural modifiers that govern grain-boundary chemistry, fracture behavior, and ultimately ballistic performance. Experimental studies demonstrate that the effectiveness of a given additive system is determined by its dominant mechanism of action rather than by its chemical composition alone.

One of the most effective mechanisms for enhancing the densification of SiC ceramics is the formation of a transient liquid phase during sintering. Oxide-based eutectic systems such as MnO–Al_2_O_3_–SiO_2_ promote viscous flow, particle rearrangement, and pore filling at temperatures significantly lower than those required for solid-state sintering. Abilev et al. have shown that ultrasound-assisted liquid-phase sintering using such eutectic additives reduces the sintering temperature of SiC ceramics to approximately 1800 °C, while achieving dense microstructures with equiaxed grains and controlled porosity [118]. The resulting materials exhibit a flexural strength of about 440 MPa and microhardness close to 30 GPa, values that meet the requirements for armor applications. These results confirm that liquid-phase-forming additives primarily act by accelerating densification and homogenizing the microstructure, rather than by strengthening the SiC matrix itself.

Beyond densification, the suppression of excessive grain growth is critical for maintaining high hardness and strength in armor ceramics. This effect is particularly pronounced when oxide nano-additives are employed. Due to their high surface energy and enhanced chemical reactivity, nano-sized oxides significantly lower the activation energy of sintering processes while simultaneously inhibiting grain coarsening.

Spark plasma sintering of SiC ceramics with oxide nano-additives has demonstrated that near-theoretical density can be achieved at ~2000 °C with minimal grain growth, resulting in a fine-grained and homogeneous microstructure [119]. In these materials, microhardness values reaching 35 GPa and an average flexural strength of approximately 450 MPa were reported, exceeding those typically obtained by conventional liquid-phase sintering. This indicates that nano-additives play a dual role by enhancing sintering kinetics and stabilizing fine-grained microstructures.

For armor ceramics, the modification of grain-boundary chemistry is at least as important as achieving high density. Additives that segregate at grain boundaries or form thin intergranular films can alter the fracture mode from predominantly transgranular to intergranular, promoting crack deflection and branching.

Microstructural observations of liquid-phase-sintered SiC ceramics reveal the presence of oxide-modified grain boundaries, which facilitate intergranular fracture and contribute to increased fracture toughness without a significant reduction in hardness [118]. A similar effect is observed in SPS-processed SiC ceramics, where thin and uniformly distributed grain-boundary phases associated with oxide nano-additives enhance damage tolerance and suppress catastrophic brittle failure [119]. Such grain boundary engineering is particularly advantageous for armor applications, as it increases the fracture surface area and improves energy dissipation during high-velocity impact.

**Figure 8:**
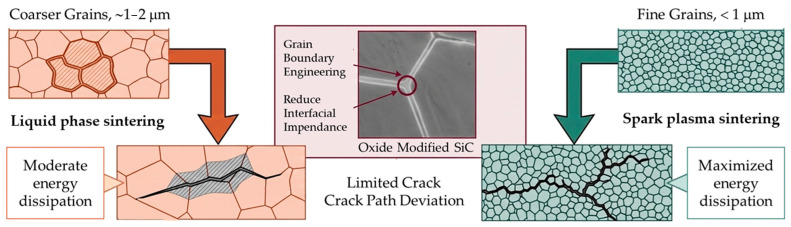
Comparative scheme of LPS and SPS SiC microstructures and crack propagation.

Importantly, the most effective additive systems for armor-grade SiC ceramics operate through synergistic mechanisms. For example, eutectic oxide systems simultaneously promote liquid-phase densification and grain-boundary modification, while nano-additives combine enhanced sintering activity with grain-growth inhibition (Figure 8).

Experimental evidence shows that ceramics produced using these combined approaches exhibit a superior balance between hardness and fracture toughness compared to materials sintered using single-mechanism additives. This synergy explains why liquid-phase sintering and SPS with carefully selected oxide additives are consistently identified as the most promising routes for advanced SiC armor ceramics.

## 5. Future Prospects

Over the past five decades, hard armor plates have evolved significantly, driven by the demand for lighter, more mobile protection. While early systems relied on heavy steel, advancements in ceramics like SiC and B_4_C, combined with polymer backings, enabled weight reduction without compromising performance. Post-2010, armor design shifted toward compact, stand-alone and modular systems, matching vest sizes to enhance mobility. Since 2015, the use of ultra-high-molecular-weight polyethylene has significantly reduced armor weight, even against steel-cored threats. This enabled the development of stacked systems: two-layer setups with an ultra-high-molecular-weight polyethylene base and a ceramic-rich top layer, offering advanced protection against armor-piercing rounds. However, these systems present logistical and durability challenges, particularly in ensuring proper fit and crack resistance in the ceramic component [5,6,7,31].

Despite advancements, with design, compositions, and the fibers used, ceramics remain the critical component for absorbing ballistic energy, with future improvements expected to be incremental.

Among armor ceramics, SiC remains one of the most promising ceramic materials for advanced armor systems due to its outstanding combination of hardness, low density, and thermal stability [4,7,154,155]. There has been a vast advancement in manufacturing SiC ceramic composites with enhanced mechanical properties over the last decades [8]. This review has highlighted significant progress in sintering techniques and use of additives in order to enhance mechanical properties.

Looking forward, several aspects need to be further researched to enhance the understanding and fulfill the application of SiC ceramics in a variety of armor types.

Recent advances in additive manufacturing, including 3D printing of SiC and SiC-based composites, offer promising routes for fabricating complex geometries and tailored microstructures that are difficult to achieve with conventional processing [156,157,158,159,160,161,162]. Techniques such as direct ink writing, fused deposition modeling, and precursor infiltration have enabled precise control over the particle orientation, fiber distribution, and layer architecture, which can enhance the mechanical performance, fracture toughness, and ballistic efficiency of printed components [156,157,158,159,160,161,162]. For example, laminated C_f_/SiC ceramics produced by 3D printing have demonstrated bending strengths of approximately 446 MPa and fracture toughness values of ~6.0 MPa·m^1/2^, indicating a competitive mechanical performance [160]. In addition, printed SiC components can achieve high hardness and relatively low density, while enabling complex architectures such as lattice or layered structures that may improve energy absorption and structural efficiency [156,157,158,159,160,161,162].

Despite these advances, challenges remain, including achieving full densification, avoiding surface oxidation during printing, ensuring reproducibility, and scaling production for practical armor applications. Additional issues such as sintering shrinkage and residual porosity require careful control of debinding, infiltration, and high-temperature sintering processes. Further research into powder handling, post-processing heat treatments, and microstructural optimization is needed to fully realize the potential of 3D-printed SiC ceramics in next-generation armor systems.

To address some of these limitations, recent studies have explored the combination of additive manufacturing with advanced sintering techniques such as SPS to produce functionally graded ceramic structures with improved densification and microstructural control [163,164,165]. Additive manufacturing enables the spatial control of the composition and microstructure, allowing the fabrication of graded architectures in which a hard ceramic-rich surface is combined with tougher or more compliant layers. For example, multi-material additive manufacturing approaches have demonstrated the ability to fabricate compositionally graded carbide ceramics with a controlled phase distribution and improved damage tolerance [163]. In addition, functionally graded SiC–B4C ceramics densified by SPS have shown enhanced mechanical properties, with reported microhardness values up to 26.2 GPa and fracture toughness 4.36 MPa·m^1/2^, depending on the composition gradient and sintering conditions [164]. Such graded structures can reduce stress concentrations during impact and improve energy dissipation compared with homogeneous ceramics. Furthermore, recent studies on graded ceramic armor concepts highlight the potential of additive manufacturing to produce architectured protective systems with an optimized hardness–toughness balance and improved resistance to crack propagation [165].

Recent advancements in armor ceramics for personnel protection focus on reducing weight while improving performance [5,6,7,31,166]. Key approaches include optimizing the composition and structure of monolithic ceramics to enhance energy dissipation, and developing ceramic composites (ceramic–ceramic, ceramic–polymer and ceramic–metal) to increase toughness and limit crack growth [4,31,167,168]. Composite systems—such as laminated or ceramic–particulate designs—are also being refined, often supported by optimized backing materials [32,168].

Improved structural properties can be achieved by tailoring particle size distributions, using directional crystallization, or creating multiphase ceramics for better ballistic resistance. Lightweight monolithic materials, such as carbide- and nitride-based ceramics (e.g., SiC—B_4_C, SiC–Si_3_N_4_, SiC–AlN, etc.), may be engineered as homogeneous or reinforced structures with whiskers, platelets, nanotubes, or mixed grain sizes to enhance energy absorption and multi-hit capability [169,170,171,172].

Another improvement lays in finding ways to use cost-effective raw materials, including industrial by-products. Manufacturing innovation is another priority, aiming to boost productivity and cut costs. Promising techniques include pressure casting, SPS, laminated structure fabrication, and porous ceramic infiltration [32,169,173,174,175,176]. Reaction bonding, including biomorphic reaction-bonded SiC ceramics, also offers a cost-effective route to high-performance armor with good multi-hit resistance [177,178,179,180].

In addition, it is essential to incorporate fundamental scientific principles and theoretical modeling to gain a deeper understanding of the microstructural evolution, phase transformations, interfacial phenomena, and densification mechanisms during the sintering of SiC ceramics [14,16,17,18,38,138]. Such comprehensive insights enable the development of generalized frameworks and predictive approaches for optimizing sintering conditions, tailoring material properties, and enhancing the performance of SiC-based ceramics in advanced armor systems.

Future development of SiC ceramics for advanced armor applications is intended to benefit from a multilayered approach that combines innovative sintering technologies, intelligent additive selection, and post-processing strategies.

A futuristic direction in the consolidation of SiC nanoceramics involves controlled powder handling in inert or regulated atmospheres and dispersion in organic media to prevent surface oxidation, a key barrier to full densification.

Post-sintering heat treatments can significantly enhance densification and mechanical properties, particularly through the initiation of α-SiC grain elongation and growth [43,83,89,141,142,143,144]. This evolution of elongated, plate-like grains contributes to toughening mechanisms such as crack bridging, deflection, and grain pull-out, with reported improvements in fracture toughness. However, an excessive heat treatment duration can lead to intergranular fracture, cavity formation, and overall degradation in mechanical performance due to over-annealing, necessitating precise thermal control to optimize results [43,83,89,141,142,143,144].

Beyond static material evaluation, the elevated-temperature performance of SiC ceramics must be examined under a wide range of thermal shock and thermo-mechanical cycling conditions that closely replicate real-world service environments. This includes integration with structural and engine-scale testing to verify the reliability of SiC-based systems under practical loading conditions [181,182].

The incorporation of tailored additives, including different combinations of compounds described in Section 4 of this review, shows promise for overcoming SiC’s inherent brittleness and improving its fracture toughness and damage tolerance. Moreover, functionally graded composites and hybrid architectures could provide improved energy absorption and multi-hit resistance, critical for modern armor systems.

In parallel, SiC-B_4_C composite ceramics have emerged as promising alternatives to monolithic B_4_C or SiC, offering a favorable balance of hardness, wear resistance, and toughness [183]. While their tribological performance under dry and water-lubricated conditions is commendable, improvements under high-load applications remain a priority, with research efforts focusing on enhancing fracture toughness to overcome brittleness [31].

Furthermore, discrepancies in reported mechanical property values highlight the urgent need for standardized testing protocols and comprehensive data correlation across studies. The future of SiC and its composites lies in integrated material–process design strategies that combine experimental methods, computational material design modeling, and in situ characterization during sintering to predict and control local and global failure mechanisms. Such an approach will ultimately lead to the next generation of high-performance, lightweight ceramic systems tailored for protective applications across defense, aerospace, and other extreme service environments.

The ballistic performance of ceramic materials is influenced by various factors, including density, porosity, hardness, fracture toughness, Young’s modulus, sonic velocity, and mechanical strength. No single property directly determines performance due to the complex and rapid nature of crack formation during impact [3,4]. Ceramics with specially tailored grain sizes or multiple crystalline phases can enhance ballistic performance without necessarily improving mechanical properties. Effective armor ceramics must balance high hardness for projectile resistance with controlled crack propagation. However, achieving both is challenging.

Effective ceramic armor design requires the consideration of all relevant physical properties, microstructure, energy dissipation capacity, and optimized manufacturing. Both dense, homogeneous ceramics and tailored heterogeneous materials may show an excellent ballistic performance [3,4]. Lightweight, cost-effective, multi-hit systems can be achieved by leveraging the fracture behavior of individual components. There is no universally optimal ceramic; material and system selection must be tailored to specific ballistic threats, manufacturing constraints, weights, and cost requirements.

## 6. Conclusions

The present review summarizes the key factors governing the densification behavior, microstructural evolution, and mechanical performance of SiC ceramics processed via different sintering routes. Particular attention is given to the role of sintering additives, reinforcing phases, and advanced processing techniques in tailoring the properties of SiC-based materials for high-performance applications:

- The mechanical properties of SiC ceramics are governed by the combined influence of powder characteristics, sintering methods, additives, and reinforcing phases.
- The incorporation of sintering additives and reinforcing phases significantly enhances densification and mechanical performance. Common effective additives include Al_2_O_3_, Y_2_O_3_, MgO, CaO, and rare-earth oxides, while TiB_2_ and TiC are widely used as reinforcing phases.
- Fine particle size and homogeneous distribution of additives promote improved densification and microstructural uniformity, leading to enhanced mechanical properties.
- Among sintering techniques, SPS provides the highest densification and improved control over grain growth due to rapid heating and short dwell times.
- Optimal properties require a balance between densification and grain growth. Moderate grain growth contributes to improved fracture toughness through crack deflection mechanisms, whereas excessive grain coarsening reduces strength and hardness.
- Variability in reported mechanical properties is often associated with differences in measurement techniques, instrumentation, and experimental conditions; therefore, such data should be interpreted comparatively.
- Advanced SiC ceramics are promising materials for demanding applications, including ballistic armor, high-temperature structural components, and wear-resistant systems.
- In armor applications, performance is governed by the synergistic interaction between densification, grain growth control, and grain boundary engineering.
- Additives that promote transient liquid-phase formation and modify grain boundary chemistry enable a transition from transgranular to intergranular fracture, thereby improving energy dissipation.
- Oxide eutectic systems and nanoscale additives are particularly effective for tailoring microstructure-driven performance.
- Mechanical properties should be evaluated together with microstructural characteristics, including grain size distribution, grain boundary phases, and fracture mode.
- Optimal performance is achieved by maintaining a balance between hardness and damage tolerance rather than maximizing a single property.
- Future research directions include the development of scalable and cost-effective processing routes, the use of advanced sintering techniques such as SPS, the design of functionally graded materials, the integration of additive manufacturing, and the application of computational modeling and machine learning for process optimization.

Overall, the performance of SiC ceramics is determined by the combined influence of processing conditions, microstructure, and phase composition. Future progress in this field will depend on integrated approaches that combine advanced sintering technologies, nanoscale additive engineering, and data-driven design strategies, enabling the development of next-generation SiC-based ceramics with improved reliability, performance, and scalability.

## Figures and Tables

**Table 1:** Various commercially produced ceramics for armor application with important properties.

Material Based on SiC	Density (g/cm^3^)	Strength (MPa)	Fracture Toughness (MPa·m^1/2^)	Young’s Modulus (GPa)	Hardness (GPa)	Ref.
CoorsTek, SiC-N, PAD	3.2	570	5.0	460	23.5	
MCC, RSSC	3.1	504	3.6–5.0	399	24.5	[31]
MCC (Aus)	3.2	486	4.0	453	30.3	
RSSC	3.2	634	4.3	450	22.6	
SiC-N	3.1	380	4.6	410	27.5–29.9	
Ceralloy 146-3E	3.1	455	4.3	420	29.9	
Hexoloy	3.2	480	3.0–4.0	410	27.5	
Purebide 5000	3.2	351	4.0	424	28.5	
SC-DS	3.2	372	5.7	425	30.7	
MCT SSS	3.2	612	6.4	453	29.7	
MCT LPS	3.0	260	4.0	359	23.0–25.0	
Ekasic-T	3.0	260	4.0	380	19.0	
SSC-702	3.1	271	4.0	407	25.5	[7]
SSC-802	3.1	200	4.0	410	21.0	
SSC-902	3.2	260	3.5	398	28.0	[10]
Self-bonded SiC	3.0–3.2	414	4.2	390	26.0	[32]

**Table 2:** Comparison of sintering strategies for SiC armor ceramics.

Sintering Strategy	Typical Temperature (°C)	Pressure (MPa)	Additive System	Relative Density (%)	Microstructural Features	Relevance to Armor Applications
Solid-state sintering (SSS)	2100–2300	–	B, C	90–95	Coarse grains, residual porosity	Limited use, insufficient toughness
Hot pressing (HP)	1900–2100	20–40	Al_2_O_3_–Y_2_O_3_	97–99	Dense, moderate grain growth	Proven armor ceramics, high cost
Liquid-phase sintering (LPS)	1750–1900	–	Al_2_O_3_–Y_2_O_3_, MnO–Al_2_O_3_–SiO_2_	96–99	Equiaxed grains, clean boundaries	Good toughness and damage tolerance
Spark plasma sintering (SPS)	1800–2000	30–80	Oxide nano-additives	≥99	Fine-grained, homogeneous	High potential for advanced armor

**Table 3:** Functional classification of sintering additives for SiC armor ceramics.

Additive Category	Typical Additives	Primary Function	Effect on Armor-Relevant Properties
Diffusion activators	B, C	Enhance solid-state diffusion	Higher density, low toughness
Liquid-phase formers	Al_2_O_3_–Y_2_O_3_, MnO–Al_2_O_3_–SiO_2_	Promote viscous flow and pore filling	Improved densification and toughness
Nano-oxide modifiers	Al_2_O_3(nano)_, MnO_(nano)_	Suppress grain growth	Increased hardness and strength
Grain-boundary modifiers	Y_2_O_3_, REE oxides	Modify interfacial energy	Crack deflection, higher K_IC_

**Table 4:** Correlation between the sintering strategy, fracture behavior, and armor performance based on reported literature trends.

Sintering Strategy	Dominant Fracture Mode	Grain-Boundary Character	Implications for Armor Performance	Ref.
SSS	Transgranular	Clean, strong boundaries	Low energy dissipation (~300–500 MPa flexural strength, ~2.5–4.0 MPa·m^1/2^ fracture toughness, ~20–25 GPa hardness)	[45,53,68,73]
HP	Mixed	Moderate intergranular phase	Balanced hardness and toughness (~500–800 MPa flexural strength, ~3.5–7.0 MPa·m^1/2^ fracture toughness, ~15–25 GPa hardness)	[32,93,96,97,99]
LPS	Intergranular	Oxide-modified boundaries	Enhanced crack deflection (~350–700 MPa flexural strength, ~4.0–7.0 MPa·m^1/2^ fracture toughness, ~20–30 GPa hardness)	[11,28,29,76,77,78,79,118]
SPS	Intergranular + crack branching	Thin, uniform boundary films	Superior damage tolerance (~500–800 MPa flexural strength, ~3.5–6.0 MPa·m^1/2^ fracture toughness, ~25–35 GPa hardness)	[112,113,114,115,116,117,119]

**Table 5:** Mechanical properties of SiC ceramics sintered without and with boron, carbon or metal additives.

Main Phase, Type of SiC	Additives	Sintering Method, Pressure (MPa), Temperature (°C)	Strength (MPa)	Fracture Toughness (MPa·m^1/2^)	Hardness (GPa)	Ref.
SiC ~55 nm	-	UHPS, 5000 MPa, 1500 °C	-	2.6	27.2	[9]
		UHPS, 15,000 MPa, 1500 °C	-	3.8	32.8	
		UHPS, 25,000 MPa, 1400 °C	-	3.6	24.8	
		UHPS, 25,000 MPa, 1500 °C	-	4.8	32.3	
SiC ~450 nm		SPS, 80 MPa, 1950 °C	-	3.6	31.9	[25]
α-SiC ~600 nm	0.5 wt% B + 3.0 wt% C	PLS, 2200 °C	341	3.1	23.3	[111]
		PLS 2-step, 2100 > 2050 °C	556	3.4	24.4	
β-SiC ~30 nm	-	SPS, 130 MPa, 1850 °C	200	2.2	-	[52]
β-SiC ~80 nm	-	SPS, 130 MPa, 1950 °C	390	2.6	-	
β-SiC ~145 nm	0.5 wt% B	SPS, 130 MPa, 1650 °C	475	3.2	-	
β-SiC ~550 nm	0.5 wt% B	SPS, 100 MPa, 1750 °C	455	3.5	-	
SiC ~420 nm	0.3 wt% B + 0.3 wt% C	SPS, 50 MPa, 2000 °C	332	3.3	20.1	[123]
SiC <1000 nm	0.5 wt % C + 0.4 wt% B	PLS, 2500 °C	359	3.1	32.0	[125]
	~7.8 wt% Mg + ~0.2 wt% Al	HP, 30 MPa, 1300 °C	172	4.6	6.7	[96]

**Table 6:** Mechanical properties of SiC ceramics sintered with oxide additives.

Main Phase, Type of SiC	Additives	Sintering Method, Pressure (MPa), Temperature (°C)	Strength (MPa)	Fracture Toughness (MPa·m^1/2^)	Hardness (GPa)	Ref.
0% α-SiC + 83.8% β-SiC	7 wt% Al_2_O_3_ + 9.2 wt% Y_2_O_3_	PLS, 1950 °C	321	6.8		[58]
8.4% α-SiC + 75.4% β-SiC			371	5.3		
25.1% α-SiC + 58.7% β-SiC			384	4.5		
50.3% α-SiC + 33.5% β-SiC			426	4.3		
83.8% α-SiC + 0% β-SiC			477	4.4		
α-SiC ~600 nm	3.1 wt% Al_2_O_3_ + 1.9 wt% Y_2_O_3_	PLS, 1950 °C	~650	~6.5	~18.8	[79]
	6.3 wt% Al_2_O_3_ + 3.7 wt% Y_2_O_3_		~615	~6.8	~18.4	
	9.4 wt% Al_2_O_3_ + 5.6 wt% Y_2_O_3_		~525	~6.4	~17.8	
	15.6 wt% Al_2_O_3_ + 9.4 wt% Y_2_O_3_		~500	~5.5	~16.2	
	3.1 wt% Al_2_O_3_ + 1.9 wt% Y_2_O_3_	PLS, 2000 °C	~565	~7.2	~20.1	
	6.3 wt% Al_2_O_3_ + 3.7 wt% Y_2_O_3_		~555	~7.5	~19.5	
	9.4 wt% Al_2_O_3_ + 5.6 wt% Y_2_O_3_		~500	~7.4	~19.0	
	15.6 wt% Al_2_O_3_ + 9.4 wt% Y_2_O_3_		~480	~6.3	~17.0	
β-SiC	6 wt% Al_2_O_3_ + 4 wt% Y_2_O_3_	PLS, 1875 °C	~532	~5.6	~21.0	[85]
89 wt% β-SiC + 1 wt% α-SiC	4.3 wt% Al_2_O_3_ + 5.7 wt% Y_2_O_3_	HP, 25 MPa, 1800 °C > PLS, 1950 °C		5.9		[82]
88 wt% β-SiC	5.2 wt% Y_3_Al_5_O_12_ + 6.8 wt% SiO_2_	HP, 25 MPa, 1800 °C > PLS, 1950 °C		6.6		
90 wt% β-SiC	5.7 wt% Al_2_O_3_ + 3.3 wt% Y_2_O_3_ + 1 wt% CaO	HP, 25 MPa, 1800 °C > PLS, 1950 °C		8.4		
0 wt% α-SiC + 93% β-SiC	2.8 wt% Al_2_O_3_ + 4.2 wt% Y_2_O_3_	HP, 30 MPa, 1950 °C > PLS, 1950 °C	~540 > 460	~3.6 > 3.8		[83]
4.6% α-SiC + 88.4% β-SiC			~530 > 455	~3.7 > 3.9		
9.3% α-SiC + 83.7% β-SiC			~545 > 425	~3.6 > 4.6		
18.6% α-SiC + 74.4% β-SiC			~560 > 450	~3.8 > 5.0		
93% α-SiC + 0% β-SiC			~710 > 605	~3.6 > 4.2		
β-SiC ~500 nm	9 wt% MgO + Al_2_O_3_ + Y_2_O_3_	HP, 25 MPa, 1800 °C	336.7	3.8	21.6	[137]
β-SiC ~500 nm + 5 vol% β-SiC ~70 nm			352.7	3.5	20.2	
β-SiC ~500 nm + 10 vol% β-SiC ~70 nm			360.8	3.5	22.5	
β-SiC ~500 nm + 15 vol% β-SiC ~70 nm			360.4	3.8	18.4	
α-SiC ~450 nm	2 mol% Y_3_Al_5_O_12_	HP, 50 MPa, 1850 °C > annealing 1950 °C 5 h		3.2		[118]
95 vol% α-SiC ~450 nm + 5 vol% β-SiC ~430 nm				~3.5		
80 vol% α-SiC ~450 nm + 20 vol% β-SiC ~430 nm				~3.7		
50 vol% α-SiC ~450 nm + 50 vol% β-SiC ~430 nm				~4.2–6.0		
20 vol% α-SiC ~450 nm + 80 vol% β-SiC ~430 nm				~3.8		
β-SiC ~430 nm				~3.8		
β-SiC ~300 nm	3.2 vol% Al_2_O_3_ + 1.8 vol% La_2_O_3_	HP, 40 MPa, 1800 °C > annealing 1950 °C 5 h.	504 > 661	4.2 > 5.5		[88]
	3.3 vol% Al_2_O_3_ + 1.7 vol% Nd_2_O_3_		550 > 620	3.9 > 5.5		
	3.3 vol% Al_2_O_3_ + 1.7 vol% Y_2_O_3_		587 > 655	3.7 > 4.6		
	3.5 vol% Al_2_O_3_ + 1.5 vol% Yb_2_O_3_		652 > 715	3.4 > 3.9		
β-SiC ~300 nm	14.9 wt% Al_2_O_3_ + 11.2 wt% Y_2_O_3_	GP, <2 MPa, 1950 °C, Ar	630	3.3	17.6	[122]
		GP, <2 MPa, 1950 °C, N_2_	870	2.2	20.7	
α-SiC (~1000 nm)	3 wt% Al_2_O_3_ + 2 wt% Y_2_O_3_	PLS, 1950 °C	332	4.9		[80]
	6 wt% Al_2_O_3_ + 4 wt% Y_2_O_3_		455	5.9		
	9 wt% Al_2_O_3_ + 6 wt% Y_2_O_3_		470	5.6		
	12 wt% Al_2_O_3_ + 8 wt% Y_2_O_3_		498	5.6		
	6 wt% Al_2_O_3_ + 9 wt% Y_2_O_3_		470	4.9		
	8 wt% Al_2_O_3_ + 12 wt% Y_2_O_3_		450	5.2		
	4.2 wt% Al_2_O_3_ + 4.4 wt% Y_2_O_3_ + 1.4 wt% MgO		440	5.2		
	1.2 wt% Al_2_O_3_ + 6.1 wt% Y_2_O_3_ + 2.7 wt% MgO		377	4.8		
α-SiC 800 nm	1.2 vol% Al_2_O_3_ + 0.94 vol% Y_2_O_3_	HP, 39 MPa, 1950 °C	565	5.4		[42]
αa-SiC 30 nm + α-SiC 800 nm (1/3 ratio)	1.2 vol% Al_2_O_3_ + 1.2 vol% Y_2_O_3_		812	6.0		
α-SiC 30 nm +α-SiC 330 nm (1/3 ratio)	2.0 vol% Al_2_O_3_ + 1.6 vol% Y_2_O_3_		548	4.9		
α-SiC ~550 nm	10 vol.% Al_2_O_3_/La_2_O_3_ (5/3 ratio)	PLS, 1950 °C		4.4	17.2	[87]
	10 vol.% Al_2_O_3_/Nd_2_O_3_ (5/3 ratio)			4.2	18.7	
	10 vol.% Al_2_O_3_/Yb_2_O_3_ (5/3 ratio)			4.0	21.4	
β-SiC 80 nm	20 wt% Al_2_O_3_/TiO_2_ (ratio 1/1)	SPS, 50 MPa, 1800 °C		3.6	18.3	[115]
β-SiC 60–80 nm	5 wt% Al_2_O_3_/TiO_2_ (ratio 1/1)			2.2	16.0	
	10 wt% Al_2_O_3_/TiO_2_ (ratio 1/1)			3.4	19.8	
	20 wt% Al_2_O_3_/TiO_2_ (ratio 1/1)			3.6	18.6	
α-SiC ~500 nm	3.06 wt% Al_2_O_3_ + 3.94 wt% Y_2_O_3_ + 5 wt% TiO_2_	PLS, 1920 °C	516	4.6	27.4	[133]
α-SiC	7 wt% Al_2_O_3_/Lu_2_O_3_ (5/3 ratio)	PLS, 1920 °C	565	4.1	27.6	[29]
	7 wt% Al_2_O_3_/Er_2_O_3_ (5/3 ratio)	PLS, 1880 °C	523	4.2	25.2	
	7 wt% Al_2_O_3_/Ce_2_O_3_ (5/3 ratio)	PLS, 1840 °C	437	4.6	23.0	
α-SiC 700 nm	5 vol% Al_2_O_3_	SPS, 40 MPa, 1700 °C		4.6	26.4	[25]
		SPS, 40 MPa, 1750 °C		4.7	26.7	
		SPS, 40 MPa, 1800 °C		5.8	26.9	
		SPS, 80 MPa, 1700 °C		5.7	26.2	
		SPS, 80 MPa, 1750 °C		5.5	28.6	
		SPS, 80 MPa, 1800 °C		5.9	28.9	
α-SiC 500 nm	3.6 wt% Al_2_O_3_ + 2.4 wt% Y_2_O_3_	PLS, 1920 °C	586	4.7	25.3	[86]
β-SiC 500 nm	5 wt% Al_2_O_3_ + 2.8 wt% Y_2_O_3_ + 0.7 wt% MgO + 0.5 wt% CaO	PLS, 1750 °C	220	4.8	23.0	[11]
		PLS, 1800 °C	347	5.2	29.3	
		PLS, 1850 °C	325	5.6	27.7	
		PLS, 1900 °C	278	6.0	26.3	
α-SiC 300 nm	4.3 wt% Al_2_O_3_ + 5.7 wt% Y_2_O_3_ + 2.5 wt% Cr_2_O_3_	PLS, 1900 °C		~5.1	~23.2	[135]
	4.3 wt% Al_2_O_3_ + 5.7 wt% Y_2_O_3_ + 5 wt% Cr_2_O_3_			~6.2	~28.2	
	4.3 wt% Al_2_O_3_ + 5.7 wt% Y_2_O_3_ + 7.5 wt% Cr_2_O_3_			~5.8	~25.0	
	4.3 wt% Al_2_O_3_ + 5.7 wt% Y_2_O_3_ + 10 wt% Cr_2_O_3_			~5.3	~24.8	
α-SiC 300 nm	4.3 wt% Al_2_O_3_ + 5.7 wt% Y_2_O_3_	PLS, 1900 °C	454	~4.3	~22.5	[30]
	4.3 wt% Al_2_O_3_ + 5.7 wt% Y_2_O_3_ + 1.5 wt% TiO_2_		471	~4.5	~23.5	
	4.3 wt% Al_2_O_3_ + 5.7 wt% Y_2_O_3_ + 3.0 wt% TiO_2_		494	~5.3	~25.0	
	4.3 wt% Al_2_O_3_ + 5.7 wt% Y_2_O_3_ + 4.5 wt% TiO_2_		523	~6.1	~27.1	
	4.3 wt% Al_2_O_3_ + 5.7 wt% Y_2_O_3_ + 6.0 wt% TiO_2_		504	~5.5	~25.7	
	4.3 wt% Al_2_O_3_ + 5.7 wt% Y_2_O_3_ + 7.5 wt% TiO_2_		487	~4.9	~24.0	
	4.3 wt% Al_2_O_3_ + 5.7 wt% Y_2_O_3_ + 9.0 TiO_2_		470	~4.1	~23.4	
	4.3 wt% Al_2_O_3_ + 5.7 wt% Y_2_O_3_ + 12.0 TiO_2_		456	~3.8	~22.8	
α-SiC 500 nm uniform-layered	8 wt% Al_2_O_3_/Y_2_O_3_ (mol. ratio 3/2)	HP, 40 MPa, 1840–1900 °C	348	7.4	22.0	[32]
α-SiC 500 nm gradual-layered	8 wt% Al_2_O_3_/Y_2_O_3_ (mol. ratio 3/2)		396	6.8	22.0	
α-SiC 500 nm pure-matrix	8 wt% Al_2_O_3_/Y_2_O_3_ (mol. ratio 3/2)		640	6.9	22.0	
SiC 150 nm	2.5 wt% MnO + 2.0 wt% Al_2_O_3_ + 4.5 wt% SiO2	UHPS, 5000 MPa, 1800 °C	400		28.0	[135]
		UHPS, 5000 MPa, 1800 °C (pre-ultrasound treatment)	440		30.0	
57 wt% SiC 280 nm + 31 wt% SiC 61,576 nm	5.5 wt% MnO + 2.0 wt% Al_2_O_3_ + 4.5 wt% SiO_2_	SPS, 19 MPa, 2000 °C	457	3.6	38.0	[136]
47 wt% SiC 280 nm + 41 wt% SiC 61,576 nm			450		31.0	
37 wt% SiC 280 nm + 51 wt% SiC 61,576 nm			454		35.0	
α-SiC 170 nm	5 wt% Al_2_O_3_/Y_2_O_3_ (ratio 1/1)	HP, 30 MPa, 1900 °C	383	4.5	14.9	[97]
		HP, 30 MPa, 1950 °C	675	5.6	17.9	
		HP, 30 MPa, 2000 °C	451	4.8	15.7	
	10 wt% Al_2_O_3_/Y_2_O_3_ (ratio 1/1)	HP, 30 MPa, 1900 °C	379	4.5	14.7	
		HP, 30 MPa, 1950 °C	649	5.5	17.6	
		HP, 30 MPa, 2000 °C	493	4.8	16.1	

**Table 7:** Mechanical properties of SiC ceramics sintered with various reinforcing additives.

Main Phase, Type of SiC	Additives	Sintering Method, Pressure (MPa), Temperature (°C)	Strength (MPa)	Fracture Toughness (MPa·m^1/2^)	Hardness (GPa)	Ref.
β-SiC 500 nm	9 wt% Mg(NO_3_)_2_/Al(NO_3_)_3_/Y(NO_3_)_3_ (67/16/17)	HP, 25 MPa, 1800 °C	263	5.6	17.1	[138,139,140,141]
β-SiC 500 nm, 10 vol% β-SiC 70 nm	9 wt% Mg(NO_3_)_2_/Al(NO_3_)_3_/Y(NO_3_)_3_ (67/16/17)		200	5.8	11.0	
	6 wt% Mg(NO_3_)_2_/Al(NO_3_)_3_/Y(NO_3_)_3_ (67/16/17)		208	3.3	8.3	
	3 wt% Mg(NO_3_)_2_/Al(NO_3_)_3_/Y(NO_3_)_3_ (67/16/17)		201	3.0	6.6	
0.9 wt% α-SiC(UF05) + 85.6 wt% β-SiC(BF17)	10.65 wt% Y_2_O_3_ + 2.9 wt% AlN	GP, <2 MPa, 1950 °C (1–8 h)		~3.6–5.0	~21–18	[43]
4.3 wt% α-SiC(UF15) + 82.1 wt% β-SiC(BF17)				~3.8–4.7	~20–18	
4.3 wt% α-SiC(UF25) + 82.1 wt% β-SiC(BF17)				~3.7–4.7	~23–18	
4.3 wt% α-SiC(UF05) + 82.1 wt% β-SiC(BF17)				~4.2–5.9	~20–21	
8.7 wt% α-SiC(UF15) + 77.8 wt% β-SiC(BF17)				~3.7–5.9	~21–18	
α-SiC/β-SiC (mol% 10/90)	10 vol% AlN/Y_2_O_3_ (mol%40/60)	GP, 10 MPa, 1980 °C 20 h annealing	495	4.9		[142]
	10 vol% AlN/Y_2_O_3_ (mol%60/40)	GP, 10 MPa, 2010 °C 9 h annealing	485	5.9		
	7 vol% AlN/Y_2_O_3_ (mol%60/40)	GP, 10 MPa, 2020 °C 16 h annealing	520	6.1		
α-SiC/β-SiC (mol% 4/96)	10 vol% AlN/Y_2_O_3_ (mol%60/40)	GP, 10 MPa, 1980 °C 32 h annealing	480	6.5		
α-SiC ~500 nm	1 wt% Al_2_O_3_ + 2 wt% Y_2_O_3_ + 1 wt% AlN	PLS, 1900 °C	582	3.1	25.7	[143]
β-SiC ~790 nm + 1% α-SiC(FCP-15)	1.08 wt% AlN + 8.92 wt% Y_2_O_3_	PLS, 2080 °C	346	4.2	15.8	[141]
		PLS, 2080 °C > 2000 °C 4 h annealing	280	5.1	9.5	
	4.2 wt% AlN + 5.8 wt% Y_2_O_3_	PLS, 2080 °C	475	5.9	17.6	
		PLS, 2080 °C > 2000 °C 4 h annealing	533	6.7	18.2	
α-SiC	4.2 wt% Y_2_O_3_ + 2.6 wt% Sc_2_O_3_ + 1.5 wt% AlN	PLS, 1950 °C 6 h annealing	509	4.1	27.2	[144]
β-SiC			520	5.1	25.0	
0.96 wt% α-SiC ~500 nm + 95.2 wt% β-SiC ~500 nm	2.08 wt% Al_2_O_3_ + 1.535 wt% Y_2_O_3_ + 0.28 wt% AlN	PLS, 1900 °C	425	6.9		[26]
	1.53 wt% Al_2_O_3_ + 1.70 wt% Y_2_O_3_ + 0.62 wt% AlN		433	6.6		
	0.86 wt% Al_2_O_3_ + 1.9 wt% Y_2_O_3_ + 1.04 wt% AlN		399	6.5		
α-SiC ~500–1000 nm	5 wt% TiN + 4.3 wt% Al_2_O_3_ + 5.7 wt% Y_2_O_3_	PLS, 1950 °C	687	7.0		[145]
	10 wt% TiN + 4.3 wt% Al_2_O_3_ + 5.7 wt% Y_2_O_3_		512	6.6		
	15 wt% TiN + 4.3 wt% Al_2_O_3_ + 5.7 wt% Y_2_O_3_		483	6.4		
0.6 wt% α-SiC ~450 nm, 59.4 wt% β-SiC ~270 nm	30 wt% TiC + 7 wt% Al_2_O_3_ + 3 wt% Y_2_O_3_	HP, 25 MPa, 1820 °C > 1930 °C 6 h annealing		6.3		[89]
	30 wt% TiC + 5 wt% Al_2_O_3_ + 5 wt% Y_2_O_3_			6.7		
	30 wt% TiC + 3 wt% Al_2_O_3_ + 7 wt% Y_2_O_3_			7.4		
	30 wt% TiC + 7 wt% Y_2_O_3_ + 3 wt% AlN			5.9		
	30 wt% TiC + 10 wt% Oxynitride			6.5		
β-SiC (UF25) ~450 nm	4.5 wt% TiC + 11 wt% Al2O3/Y2O3 (mass ratio 3/2)	HP, 25 MPa, 2000 °C	705	6.3	18.8	[95]
	12.9 wt% TiC + 6.5 wt% Al2O3/Y2O3 (mass ratio 3/2)		550	5.9	19.1	
	24 wt% TiC + 5.8 wt% Al2O3/Y2O3 (mass ratio 3/2)		475	5.8	19.7	
α-SiC ~600 nm	20 vol% TiC (100 nm)	HIP, 200 MPa, 1850 °C	682	4.0	22.5	[104]
	20 vol% TiC (1000 nm)		674	5.1	21.7	
β-SiC ~270 nm	9.8 wt% TiC + 1 wt% Al + 0.5 wt% B + 0.5 wt% C	SPS, 40 MPa, 1800 °C	579	4.8		[115]
	19.6 wt% TiC + 1 wt% Al + 0.5 wt% B + 0.5 wt% C		634	5.3		
	29.4 wt% TiC + 1 wt% Al + 0.5 wt% B + 0.5 wt% C		769	5.8		
	39.2 wt% TiC + 1 wt% Al + 0.5 wt% B + 0.5 wt% C		720	6.3		
β-SiC ~353 nm	15 wt% TiC	SPS, 40 MPa, 1750 °C	532	2.4	18.8	[116]
	30 wt% TiC		562	5.9	23.9	
	15 wt% TiC	SPS, 40 MPa, 1800 °C	605	3.0	24.4	
	30 wt% TiC		647	6.3	28.1	
α-SiC (FCP 15C) ~500 nm	3 wt% Al_2_O_3_ + 4 wt% Y_2_O_3_ + 5 wt% TiO_2_	PLS, 1920 °C	516	4.6	27.4	[133]
	3 wt% Al_2_O_3_ + 4 wt% Y_2_O_3_ + 5 wt% TiC		450	4.3	25.1	
α-SiC ~2000 nm	-	ESS, 45 MPa, 2000 °C, (5000A, 5 V)		2.9	2.9	[146]
	20 vol% TiC			4.3	6.6	
	40 vol% TiC			5.7	21.5	
	20 vol% VC			2.6	3.4	
	40 vol% VC			5.2	13.7	
β-SiC ~300 nm	30 vol% TiC + 1 vol% Cr_3_C_2_	HP, 30 MPa, 1950 °C	750	4.6		[93]
	30 vol% TiC + 10 vol% Cr_3_C_2_	HP, 30 MPa, 1900 °C	810	4.6		
		HP, 30 MPa, 1950 °C	670	6.5		
SiC ~600 nm	7.5 vol% TiC + 12.5 vol% TiB_2_	SPS, 80 MPa, 2000 °C	511	6.0	20.4	[44]
	15 vol% TiC + 25 vol% TiB_2_		588	6.2	22.9	
SiC ~500–1000 nm not purified	1.5 wt% B4C + 3.5 wt% C	PLS, 2000 °C	241	4.2	22.7	[41]
SiC ~500–1000 nm HF + Ultrasonic			245	5.1	26.0	
SiC ~500–1000 nm HF purified			298	5.1	26.0	
SiC ~500–1000 nm comm. 98.5% pure			315	5.0	27.0	
β-SiC	1 wt% C + 6 wt% Y_2_O_3_ + 4 wt% Al_2_O_3_	PLS, 1900 °C		2.8	9.6	[147,148]
	1 wt% B4C + 1 wt% C + 6 wt% Y_2_O_3_ + 4 wt% Al_2_O_3_			3.5	11.6	
	2 wt% B4C + 1 wt% C + 6 wt% Y_2_O_3_ + 4 wt% Al_2_O_3_			4.3	14.7	
	3 wt% B4C + 1 wt% C + 6 wt% Y_2_O_3_ + 4 wt% Al_2_O_3_			4.9	16.2	
	4 wt% B4C + 1 wt% C + 6 wt% Y_2_O_3_ + 4 wt% Al_2_O_3_			5.4	18.7	
	5 wt% B4C + 1 wt% C + 6 wt% Y_2_O_3_ + 4 wt% Al_2_O_3_			5.9	22.9	
	6 wt% B4C + 1 wt% C + 6 wt% Y_2_O_3_ + 4 wt% Al_2_O_3_			5.2	17.5	
β-SiC ~300 nm	10 vol% TiB_2_	HP, 20 MPa, 2080 °C	560	4.3		[98]
	20 vol% TiB_2_		490	5.0		
	30 vol% TiB_2_		540	5.5		
β-SiC ~750 nm	5 vol% TiB_2_	PLS, 2190 °C		3.5	30.0	[149]
	10 vol% TiB_2_			3.6	27.0	
	15 vol% TiB_2_			3.9	23.0	
α-SiC ~400 nm	5% vol TiB_2_	PLS, 2000 °C		4.9		[150]
	10% vol TiB_2_			5.7		
	15% vol TiB_2_			5.8		
	20% vol TiB_2_			5.9		
	15% vol TiB_2_	PLS, 2100 °C		6.3		
β-SiC	7 wt% Al_2_O_3_ + 3 wt% Y_2_O_3_	HP, 25 MPa, 1850 °C	611	3.6		[92]
	30 wt% TiB_2_ + 7 wt% Al_2_O_3_ + 3 wt% Y_2_O_3_	HP, 25 MPa, 1850 °C	571	4.4		
		HP, 25 MPa, 1850 °C > 1950 °C 6 h annealing	550	6.7		
		HP, 25 MPa, 1850 °C > 1950 °C 12 h annealing	501	6.4		
β-SiC ~700 nm	12 vol% TiB_2_ + 4.3% Al_2_O_3_ + 5.7% Y_2_O_3_	PLS, 1940 °C	485	4.3	17.0	[151]
	24 vol% TiB_2_ + 4.3% Al_2_O_3_ + 5.7% Y2O3		470	5.3	16.5	
	30 vol% TiB_2_ + 4.3% Al_2_O_3_ + 5.7% Y_2_O_3_		410	5.7	13.3	
α-SiC 400–480 nm	20 vol% TiB_2_, C	HIP, 200 MPa, 2200 °C	690	6.0		[105]
	20 vol% TiB_2_, Aqueous C	HIP, 200 MPa, 2000 °C	730	4.7		
	20 vol% TiB_2_, Phenol resin	HIP, 200 MPa, 2100 °C	826	4.8		
SiC ~600 nm	20 vol% TiB_2_ + 10 vol% AlN + 1 wt% Y_2_O_3_	SPS, 40 MPa, 1900 °C	438	5.1	26.8	[27]
		SPS, 40 MPa, 1950 °C	450	5.4	26.8	
		SPS, 40 MPa, 2000 °C	553	5.7	28.3	
		SPS, 40 MPa, 2050 °C	444	5.0	27.0	
		SPS, 40 MPa, 2100 °C	467	5.0	27.1	
	20 vol% TiB_2_ + 20 vol% AlN + 1 wt% Y_2_O_3_	SPS, 40 MPa, 2000 °C		5.1	25.3	
		SPS, 40 MPa, 2050 °C		5.1	26.8	
		SPS, 40 MPa, 2100 °C		5.0	27.2	
SiC ~3310 nm	7.2 wt% Mg_2_Si + 10 wt% Ni_3_Al	SPS, 50 MPa, 1300 °C		10.4	13.6	[152,153]
	6.4 wt% Mg_2_Si + 20 wt% Ni_3_Al	SPS, 50 MPa, 1300 °C		10.1	12.7	
92 wt% β-SiC ~500 nm + 1 wt% α-Si ~450 nm	2.49 wt% SiO_2_ + 2.38 wt% Al_2_O_3_ + 1.32 wt% Y_2_O_3_ + 0.32 wt% CaCO_3_	PLS, 1850 °C	642	4.9	27.0	[28]
88 wt% β-SiC ~500 nm + 1 wt% α-SiC ~450 nm	2.36 wt% SiO_2_ + 5.48 wt% Al_2_O_3_ + 3.03 wt% Y_2_O_3_ + 0.74 wt% CaCO_3_		686	5.4	26.3	
84 wt% β-SiC ~500 nm + 1 wt% α-SiC ~450 nm	2.26 wt% SiO_2_ + 7.74 wt% Al_2_O_3_ + 4.29 wt% Y_2_O_3_ + 1.05 wt% CaCO_3_		618	4.8	24.0	
78 wt% β-SiC ~500 nm + 1 wt% α-SiC ~450 nm	2.08 wt% SiO_2_ + 11.81 wt% Al_2_O_3_ + 6.29 wt% Y_2_O_3_ + 1.56 wt% CaCO_3_		479	4.5	20.8	

## Data Availability

No new data were created or analyzed in this study. Data sharing is not applicable to this article.
